# Cryo-EM structures of the *Synechocystis* sp. PCC 6803 cytochrome *b*_6_*f* complex with and without the regulatory PetP subunit

**DOI:** 10.1042/BCJ20220124

**Published:** 2022-07-15

**Authors:** Matthew S. Proctor, Lorna A. Malone, David A. Farmer, David J.K. Swainsbury, Frederick R. Hawkings, Federica Pastorelli, Thomas Z. Emrich-Mills, C. Alistair Siebert, C. Neil Hunter, Matthew P. Johnson, Andrew Hitchcock

**Affiliations:** 1 Plants, Photosynthesis and Soil, School of Biosciences, University of Sheffield, Sheffield S10 2TN, U.K.; 2 Electron Bio-imaging Centre, Diamond Light Source, Didcot OX11 0DE, U.K.; 3 School of Biological Sciences, University of East Anglia, Norwich NR4 7TJ, U.K.

**Keywords:** cryo-EM, cyanobacteria, cytochrome *b_6_f*, PetP, photosynthesis, *Synechocystis*

## Abstract

In oxygenic photosynthesis, the cytochrome *b*_6_*f* (cyt*b*_6_*f*) complex links the linear electron transfer (LET) reactions occurring at photosystems I and II and generates a transmembrane proton gradient via the Q-cycle. In addition to this central role in LET, cyt*b*_6_*f* also participates in a range of processes including cyclic electron transfer (CET), state transitions and photosynthetic control. Many of the regulatory roles of cyt*b*_6_*f* are facilitated by auxiliary proteins that differ depending upon the species, yet because of their weak and transient nature the structural details of these interactions remain unknown. An apparent key player in the regulatory balance between LET and CET in cyanobacteria is PetP, a ∼10 kDa protein that is also found in red algae but not in green algae and plants. Here, we used cryogenic electron microscopy to determine the structure of the *Synechocystis* sp. PCC 6803 cyt*b*_6_*f* complex in the presence and absence of PetP. Our structures show that PetP interacts with the cytoplasmic side of cyt*b*_6_*f*, displacing the C-terminus of the PetG subunit and shielding the C-terminus of cytochrome *b*_6_, which binds the heme *c*_n_ cofactor that is suggested to mediate CET. The structures also highlight key differences in the mode of plastoquinone binding between cyanobacterial and plant cyt*b*_6_*f* complexes, which we suggest may reflect the unique combination of photosynthetic and respiratory electron transfer in cyanobacterial thylakoid membranes. The structure of cyt*b*_6_*f* from a model cyanobacterial species amenable to genetic engineering will enhance future site-directed mutagenesis studies of structure-function relationships in this crucial ET complex.

## Introduction

Cytochrome *b*_6_*f* (cyt*b*_6_*f*) catalyzes the rate limiting step of the photosynthetic linear electron transfer (LET) chain in plant, algal and cyanobacterial thylakoid membranes, connecting the light-driven reactions at photosystems I and II (PSI and PSII). The cyt*b*_6_*f* complex uses the so-called Q-cycle [1] to couple ET between the PSII electron acceptor plastoquinol (PQH_2_) and the PSI electron donor plastocyanin (Pc) (or cytochrome *c*_6_ in some cyanobacteria) to proton transfer across the membrane, conserving energy as a proton motive force for ATP synthesis [2,3]. The Q-cycle involves the bifurcated transfer of electrons from the PQH_2_ donor bound at the oxidizing (Q_p_) site into so-called high-potential (2Fe-2S centre, bound by the Rieske iron-sulfur protein (ISP) subunit, and heme *f,* bound by the cytochrome *f* subunit) and low-potential (hemes *b*_p_, *b*_n_ and *c*_n_, all bound by the cytochrome *b*_6_ subunit) cofactor chains. The high-potential chain transfers an electron to Pc, while the low-potential chain recycles the other electron to a plastoquinone (PQ) acceptor bound at the reducing (Q_n_) site. During the Q-cycle, protons are released to the lumen during PQH_2_ oxidation at the Q_p_ site and taken up during PQ reduction at the Q_n_ site. The crucial role of cyt*b*_6_*f* in photosynthesis has recently been demonstrated in studies that showed plant growth could be enhanced via overproduction of the ISP subunit [4,5]. Yet, despite its importance, many of the mechanistic details underlying the function of the cyt*b*_6_*f* complex remain unclear.

In addition to its central role as the major electron-proton transfer coupling site in the LET chain, cyt*b*_6_*f* also participates in a range of crucial regulatory functions, including cyclic electron transfer (CET), state transitions and photosynthetic control [2,6]. Photosynthetic control avoids over-reduction and photo-oxidative damage by regulating the rate of electron delivery to PSI, using the lumenal pH to tune the electron transfer efficiency of cyt*b*_6_*f* [2,6,7]. Conversely, other regulatory functions of cyt*b*_6_*f* are believed to be mediated through transient interactions with weakly bound auxiliary proteins. For instance, in plants and green algae the role of cyt*b*_6_*f* in state transitions is fulfilled by binding and modulation of the serine-threonine kinase Stn7/Stt7, which phosphorylates light-harvesting complex II (LHCII) to adjust the relative antenna sizes of PSI and PSII to balance their excitation rates [8–10]. In contrast, state transitions in cyanobacteria involve the movement of phycobilisome antenna between PSI and PSII in a manner independent of cyt*b*_6_*f* [11]. In plants and green algae cyt*b*_6_*f* may directly facilitate the major CET pathway through binding the ferredoxin-NADP^+^ reductase (FNR), allowing electrons from ferredoxin (Fd) to be channelled directly to the PQ pool via heme *c*_n_ [12–14]. The proton gradient regulation 5 (PGR5) protein is crucial to CET in plants and green algae [15]. PGR5 may influence FNR binding to the thylakoid [16,17] and it associates with cyt*b*_6_*f* [18,19], regulating cyt*b*_6_*f* activity under CET conditions [20]. In the green alga *Chlamydomonas reinhardtii,* PetO, a single-pass transmembrane protein, interacts with subunit IV of cyt*b*_6_*f* under conditions favouring CET and state transitions [21–23]. In contrast, the major CET pathway in cyanobacteria appears to involve the Fd-PQ reductase activity of the photosynthesis-related NADH dehydrogenase-like complex 1 (NDH-1) [24]. Nonetheless, there is evidence that cyanobacterial cyt*b*_6_*f* may also mediate a green algae/angiosperm-like PGR5-FNR-dependent CET [25,26]. A proposed key player in CET/LET regulation in cyanobacteria is the PetP protein, also found in red algae but absent from plants and green algae, which has been biochemically isolated with cyt*b*_6_*f* in both *Synechocystis* sp. PCC 6803 (hereafter *Synechocystis*) and *Thermosynechococcus elongatus* BP-1 (hereafter *T. elongatus*) [27–29]. A *T. elongatus* Δ*petP* mutant displayed a ∼30% decrease in LET, while the relative rate of CET remained virtually unchanged [30]. PetP was therefore suggested to play a role in thylakoid membrane organization, mediating interactions between cyt*b*_6_*f* and other complexes to modulate the balance of LET and CET [30], and more widely the branching of electrons between the photosynthetic and respiratory ET chains, a unique feature of cyanobacterial thylakoids [31,32]. A solution nuclear magnetic resonance (NMR) structure of PetP showed that it adopts an SH3-type fold, as seen in a range of other thylakoid proteins known to modulate ET function such as the α subunit of the Fd–thioredoxin reductase, NdhS of NDH-1 and PsaE of PSI [33].

A high-resolution structure defining the specific nature of the cyt*b*_6_*f*–PetP interaction would advance our understanding of the mechanism by which auxiliary proteins such as PetP modulate cyt*b*_6_*f* function. Here, we further our knowledge of cyt*b*_6_*f* structure, function and regulation by determining cryogenic electron microscopy (cryo-EM) structures of the cyt*b*_6_*f* complex from the extensively studied and easily transformable model cyanobacterium *Synechocystis* with and without PetP.

## Materials and methods

### Growth of *Synechocystis* and generation of the Strep-tag^®^ II-tagged PetA strain

The *Synechocystis* wild type (WT)-P substrain [34] was used in this study and was grown at 30°C on BG11 media [35] supplemented with 10 mM TES (Sigma–Aldrich)-KOH pH 8.2, 1.5% (w/v) agar and 0.3% (w/v) sodium thiosulfate. Liquid cultures for purification of protein complexes lacked sodium thiosulfate and were grown photoautotrophically with ∼100 µmol photons m^−2^ s^−1^ illumination in 8 L vessels bubbled with sterile air.

To facilitate purification of the cyt*b*_6_*f* complex, we replaced the native *petA* gene with a copy that adds a C-terminal Ser-Ala linker and Strep-tag® II (Trp–Ser–His–Pro–Gln–Phe–Glu–Lys) to the protein using a method similar to that used previously to add a His-tag [36]. A linear DNA fragment was generated from three PCR products by overlap-extension (OLE)-PCR; details of all primers used in this study are provided in Supplementary Table S1. The first fragment consisted of the 3′ end of *petA* fused to the linker-Strep-tag® II sequence followed by a 25 bp region of homology to the 5′ end of the chloramphenicol acetyl transferase (*cat*) gene (this fragment was obtained as a gBLOCK from Integrated DNA Technologies). The *cat* gene was PCR amplified from pACYCDuet^TM^-1 (Novagen) using the primers *cat*-F and *cat*-R. The third fragment comprising a ∼500 bp region homologous to the sequence downstream of *petA* was PCR amplified from the *Synechocystis* genome using primers *petA*-ds-F and *petA*-ds-R. OLE-PCR with primers *petA*-SII-F and *petA*-SII-R was used to join the three fragments and the resulting linear product was sequence verified (Eurofins) prior to introduction into WT *Synechocystis* by natural transformation. Transformants were selected on BG11 agar containing 12.5 µg ml^−1^ chloramphenicol and single colonies were picked and sequentially patched onto BG11 agar containing increasing concentrations of chloramphenicol up to 68 µg ml^−1^. The resulting PetA-StrepII strain was confirmed to be fully segregated at the *petA* locus by PCR with primers *petA*-screen-F and *petA*-screen-R and the sequence of the modified *petA* gene was verified by automated DNA sequencing (Eurofins).

### Purification and *in situ* reconstitution of cyt*b*_6_*f* and PetP

*Synechocystis* PetA-StrepII cells from a total culture volume of 32 L (4 × 8 L cultures) were harvested by centrifugation (14 334×***g***, 10 min, 4°C), resuspended in buffer A (25 mM sodium phosphate pH 7.6, 10 mM MgCl_2_, 50 mM NaCl, 10% (w/v) glycerol) and broken by bead beating with 0.1 mm glass beads (Thistle Scientific) for eight rounds of 55 s cycles with cooling on ice for 3 min between cycles. Unbroken cells were pelleted by centrifugation at 4696×***g*** for 20 min at 4°C and the supernatant was centrifuged again (48 400×***g***, 30 min, 4°C) to pellet the thylakoid membranes. Thylakoid membranes were homogenized in buffer B (25 mM sodium phosphate pH 7.6, 10 mM MgCl_2_, 50 mM NaCl) and solubilized by incubation with 1.5% (w/v) glyco-diosgenin (GDN; Anatrace) for 1 h at 4°C with gentle agitation. Following centrifugation (48 400×***g***, 30 min, 4°C), the soluble fraction (supernatant) was diluted 2-fold in buffer B before application to a 5 ml StrepTrap^TM^ HP column (Merck) equilibrated in the same buffer. The column was washed with 30 column volumes of buffer C (25 mM sodium phosphate pH 7.6, 10 mM MgCl_2_, 400 mM NaCl, 0.02% (w/v) GDN) before elution in buffer B supplemented with 2.5 mM d-Desthiobiotin (Merck). The column eluate was concentrated to ∼500 µl in a 100 kDa molecular weight cut-off (MWCO) protein spin concentrator (Amicon) and applied to a Cytiva HiLoad® 16/600 Superdex® 200 pg size exclusion chromatography column (Merck) equilibrated with buffer D (25 mM sodium phosphate pH 7.6, 10 mM MgCl_2_, 150 mM NaCl, 0.02% (w/v) GDN). Fractions containing cyt*b*_6_*f*, as identified by UV–Vis absorption spectroscopy, were pooled and concentrated to 17 µM.

The *Synechocystis petP* gene (ssr2998) was codon optimized for expression in *Escherichia coli* and cloned into the NdeI and XhoI sites of pET28a(+) (Novagen). Production of N-terminally hexa-Histidine (His)-tagged PetP (His-PetP) was performed in *E. coli* BL21(DE3) (ThermoFisher Scientific) by adding a 5 ml LB starter culture to 1 L autoinduction media (Formedium) at 37°C with 220 rpm shaking until an optical density (OD) at 600 nm of 0.6 was reached, at which point the temperature was lowered to 18°C and the culture was incubated for a further 16 h. The cells were harvested by centrifugation at 4696×***g*** for 15 min at 4°C and resuspended in buffer E (25 mM sodium phosphate pH 7, 10 mM MgCl_2_, 300 mM NaCl). Cells were supplemented with DNAse I (Sigma–Aldrich) and a cOmplete EDTA-free protease inhibitor tablet (Roche) before breakage by two passes through a chilled French pressure cell at 18 000 psi. The cell lysate was clarified by centrifugation at 75 600×***g*** for 30 min at 4°C and imidazole was added to a final concentration of 10 mM before application to a 5 ml HisTrap column (GE Healthcare) pre-equilibrated in buffer E. The column was sequentially washed with 10 volumes of buffer E supplemented with 10, 20 or 50 mM imidazole prior to elution of His-PetP with 400 mM imidazole. The eluate containing PetP was concentrated to 2.5 ml using a 3 kDa MWCO protein spin concentrator (Amicon) and buffer exchanged into buffer D using a PD-10 Desalting column (GE Healthcare).

His-PetP was re-bound to a 300 µl Ni^2+^-NTA agarose immobilized metal affinity chromatography (IMAC) column (Qiagen) and washed with 5 ml buffer D. Purified cyt*b*_6_*f* was passed through the column three times prior to washing with 10 ml buffer D, followed by elution in buffer D supplemented with 100 mM l-Histidine (Sigma–Aldrich). The eluate was concentrated to ∼100 µl and the concentration of l-Histidine was lowered by a 60-fold dilution in buffer D prior to re-concentration to ∼100 µl in a 100 kDa MWCO protein spin concentrator (Amicon). The sample was diluted to a concentration of 20 µM cyt*b*_6_*f* prior to preparation of cryo-EM grids. *Synechocystis* thylakoids were also applied to 300 µl Ni^2+^-NTA agarose IMAC columns functionalized with His-PetP with washing and elution as described above. Membrane samples applied to control columns not pre-bound with His-PetP were treated in the same way.

### SDS/BN-PAGE and detection of *c*-type cytochromes by enhanced chemiluminescence

For SDS–PAGE analysis of purified cyt*b*_6_*f*, protein samples were mixed with an equal volume of 2× Laemmli sample buffer (Merck) and boiled for 10 min prior to separation on precast NuPAGE 12% Bis-Tris gels (Invitrogen). For BN-PAGE analysis, cyt*b*_6_*f* was diluted in 4× sample buffer (100 mM Tris–HCl pH 7.5, 0.05% (w/v) bromphenol blue, 40% (w/v) glycerol) and analyzed on precast NativePAGE 3–12% Bis-Tris gels (Invitrogen). Gels were stained with Coomassie Brilliant Blue and imaged using an Amersham 600 imager (GE Healthcare). Alternatively, SDS–PAGE separated proteins were transferred to polyvinylidene fluoride (PVDF) membranes (ThermoFisher Scientific) as described previously [37] and cytochrome *c*-mediated chemiluminescence was detected using the WESTAR ETA C 2.0 chemiluminescent substrate (Cyanagen) and an Amersham Imager 600 (GE Healthcare).

### Quantification of purified dimeric cyt*b*_6_*f* using redox difference spectra

UV/Vis absorbance and redox difference spectra were recorded at room temperature on a Cary60 spectrophotometer (Agilent) as described in [38]. Briefly, for redox difference spectra hemes were first fully oxidized by the addition of a few grains of potassium ferricyanide, followed by reduction with a few grains of sodium ascorbate (cytochrome *f* heme) and then a few grains of sodium dithionite (cytochrome *f* and cytochrome *b*_6_ hemes). At each stage, the sample was mixed thoroughly and incubated for ∼1 min prior to recording the spectra. Redox difference spectra (ascorbate-reduced minus ferricyanide-oxidized and dithionite-reduced minus ascorbate-reduced) were used to determine the concentrations of the *c* heme of cytochrome *f* and the two *b* hemes of cytochrome *b*_6_ using extinction coefficients of 25 mM^−1^ cm^−1^ and 21 mM^−1^ cm^−1^, respectively [39].

### Analysis of carotenoid content of purified cyt*b*_6_*f* by reversed-phase high-performance liquid chromatography

Pigments were extracted from purified cyt*b*_6_*f* in 100% methanol and separated by reversed-phase high-performance liquid chromatography (RP-HPLC) on an Agilent 1200 HPLC system with a Phenomenex Luna C18 column (5 µm, 250 × 4.6 mm) according to the slightly modified method of Lagarde et al. [40] described by Proctor et al. [37]. Absorbance was monitored at 450 nm and 665 nm and carotenoid species and chlorophyll *a* were identified by their known absorption spectra and retention times [37].

### Cryo-EM specimen preparation and data acquisition

Pure *Synechocystis* cyt*b*_6_*f* (5 µl, 17 µM) was applied to lacey carbon-coated 300 mesh Cu grids (EM Resolutions Ltd.) after a 25 s glow discharge at 10 mA under partial vacuum. Grids were plunge frozen in liquid ethane using a Leica EM GP 2 at 70% relative humidity and 15°C with a blot time of 3 s. Data were acquired using EPU's aberration-free image shift (AFIS) automatic data collection routine with a Titan Krios G2 microscope operating at 300 kV (ThermoFisher Scientific) equipped with a K3 direct electron detector (Gatan Inc.) and an imaging filter with a 20 eV slit (Gatan Inc.). A total of 18 151 movies were collected using super-resolution mode with an effective pixel size of 0.53 Å (binned to 1.06 Å within EPU) and a total dose of 45 e^−^ Å^−2^, dose-fractionated into 45 frames. A defocus range of −1.2 µm to −2.5 µm in steps of −0.3 µm was used.

The purified *Synechocystis* cyt*b*_6_*f*–PetP complex (5 µl, 20 µM) was applied to a holey QUANTIFOIL R 1.2/1.3 carbon-coated Cu mesh 300 grid (Quantifoil Micro Tools GmbH) after a 25 s glow discharge at 10 mA under partial vacuum. After a 20 s incubation, the grid was blotted for 4 s then vitrified in liquid ethane using a Leica EM GP 2 at 15°C and 80% relative humidity. Data were acquired as above and a total of 20 133 movies were collected using super-resolution mode with an effective pixel size of 0.53 Å (binned to 1.06 Å within EPU) and a total dose of 43 e^−^ Å^−2^, dose-fractionated into 50 frames. A defocus range of −1.2 µm to −2.4 µm in steps of −0.3 µm was used.

### Image processing and 3D reconstruction

Beam-induced motion correction and dose-fractionation were carried out using MotionCor2 (v1.4.0) [41]. Contrast transfer function (CTF) parameters of the dose-weighted motion-corrected images were estimated using GCTF [42] or CTFFIND (v4.1.14) [43]. Unless otherwise stated, all subsequent processing steps were performed using RELION 3.1 [44–46].

For the cyt*b*_6_*f* complex lacking PetP 4 032 212 particles were picked from 18 151 micrographs via a semi-automated approach. The particles were extracted using a box size of 220 × 220 pixels and subjected to two rounds of reference-free 2D classification (Supplementary Figure S1B). A typical micrograph showing picked particles is shown in Supplementary Figure S1A. Particles that categorized into poorly defined classes were rejected, while the remaining 3 396 654 (84.2%) particles were used for further processing. A subset of 460 584 particles (∼11.4%) was used to generate a *de novo* initial model using the ‘3D initial model’ subroutine. The initial model low-pass filtered to 50 Å was used as a reference map for subsequent 3D classification into four 3D classes. The four low-resolution 3D classes were analyzed in ChimeraX (v 1.1.1) [47] before one 3D class (class 2, 9.32 Å, ∼24.5% of initially picked particles) was subjected to a further round of 3D classification generating six classes. Of these six 3D classes, one stable 3D class (class 2, 6.85 Å, 413 442 particles, ∼10.2%) of sufficient homogeneity was selected for high-resolution 3D auto-refinement. This subset of refined particles was then re-extracted and re-centred before another round of 3D auto-refinement was carried out. Per-particle CTF-refinement was carried out and a soft mask was created, which included the detergent shell; masked CTF-refined particles were subjected to a further round of 3D-refinement resulting in a map at 4.05 Å. The map was corrected for the modulation transfer function (MTF) of the Gatan K3 camera then further sharpened using the post-processing procedure to 3.53 Å. Particles were polished using the ‘Bayesian polishing’ subroutine in RELION, then two further rounds of CTF-refinement and post-processing were performed until there was no further improvement in the resolution. The final global resolution estimate of 3.15 Å was based on the ‘gold-standard’ Fourier shell correlation (FSC) cut-off of 0.143 (Supplementary Figure S1C). Local resolution was determined using one of two unfiltered half-maps as an input, a calibrated pixel size of 0.53 and a B-factor of −78. The output local resolution map is shown in Supplementary Figure S1D,E and the entire process is summarized in Supplementary Figure S2A.

For the cyt*b*_6_*f*–PetP complex, crYOLO (v1.8.0) [48] was used with its broadly applicable ‘general model’ to automatically pick 1 169 445 particles (Supplementary Figure S1F). The particles were extracted with a box size of 160 × 160 pixels, downsampled 2× and subjected to two rounds of 2D classification (Supplementary Figure S1G). The most appropriate classes were chosen after each round, reducing the number of particles to 313 169 (∼26% of those picked), which were used to generate a *de novo* initial model. The initial model generated was low-pass filtered to 30 Å and used as a reference for refinement of the downsampled particles, which achieved a refinement resolution closely approaching the Nyquist limit (4.34 Å vs 4.24 Å). The refined particles were re-extracted at full sampling rate and subjected to another refinement, which achieved a resolution of ∼3.30 Å after masking and sharpening. The variation within the refined particles was then assessed using 3D classification, masked and with no alignment (using the alignment parameters from the previous refinement step). A single class out of three was selected for the prevalence of high-resolution features, reducing the number of particles to 152 860 (∼13% of initially picked particles). The particles were subjected to further 3D-refinement, then a round of CTF-refinement to estimate higher-order aberrations, beam tilt and anisotropic magnification, followed by per-particle fitting of defocus and per-micrograph fitting of astigmatism and B-factor. The particles were then ‘polished’, estimating and correcting for the beam-induced motion on a per-particle basis. Following a further round of CTF-refinement as above, the final 3D-refinement resulted in a resolution of 2.80 Å. This, and all other resolution estimates referenced above, was calculated using the ‘gold-standard’ FSC of two independent half-maps, with a threshold of 0.143 (Supplementary Figure S1H). The local resolution map is shown in Supplementary Figure S1I,J and the entire process is summarized in Supplementary Figure S2B.

### Model building

For the cyt*b*_6_*f* complex lacking PetP, an initial homology-based approach was performed using the crystal structure of the *Nostoc* sp. PCC 7120 cyt*b*_6_*f* complex (PDB: 4OGQ) [49] as a template. The model was rigid-body docked into the density using the ‘fit in map’ tool in ChimeraX [47]. This was followed by manual adjustment and real-space refinement using Coot [50]. Sequence assignment and fitting was guided by bulky residues such as Arg, Trp, Tyr and Phe. After fitting of the polypeptide chains and cofactors in one-half of the dimeric complex, the other half of the complex was then independently fit into the C1 density map. After fitting of both halves of the complex, cofactors and lipids were fit into regions of unassigned density. The final model underwent global refinement and minimization using the real space refinement tool in PHENIX [51]. The final refinement statistics are summarized in Supplementary Table S2.

For the cyt*b*_6_*f* complex with PetP, the *Synechocystis* model described above was fit into the density map using USCF Chimera (v 1.13) [52] and the remaining density on the cytoplasmic side of the complex was attributed to PetP. A homology model of PetP was generated using HHPRED alignment guided modelling with MODELLER [53,54]. The model was based on the solution NMR structure of PetP from *T. elongatus* (PDB: 2N5U) [33], which aligned with a 99.94 HHPRED probability score and fit well into the remaining density with rigid fitting as above. The docked model was subject to a round of refinement with PHENIX (v 1.19.2) real-space refinement and then subsequently manually fit and refined within Coot (v 0.9.2). Ligands were added into unmodelled density and following a refinement of the model with REFMAC (v 5.8) [55] the map was locally sharpened using LocScale (v 0.1) [56]. The model was iteratively improved manually and with PHENIX until validation statistics from the PHENIX validation procedure converged between runs. The final refinement statistics are summarized in Supplementary Table S3.

## Results

### Isolation of dimeric cyt*b*_6_*f* from *Synechocystis* and reconstitution of the cyt*b*_6_*f*–PetP complex

**Figure 1.**
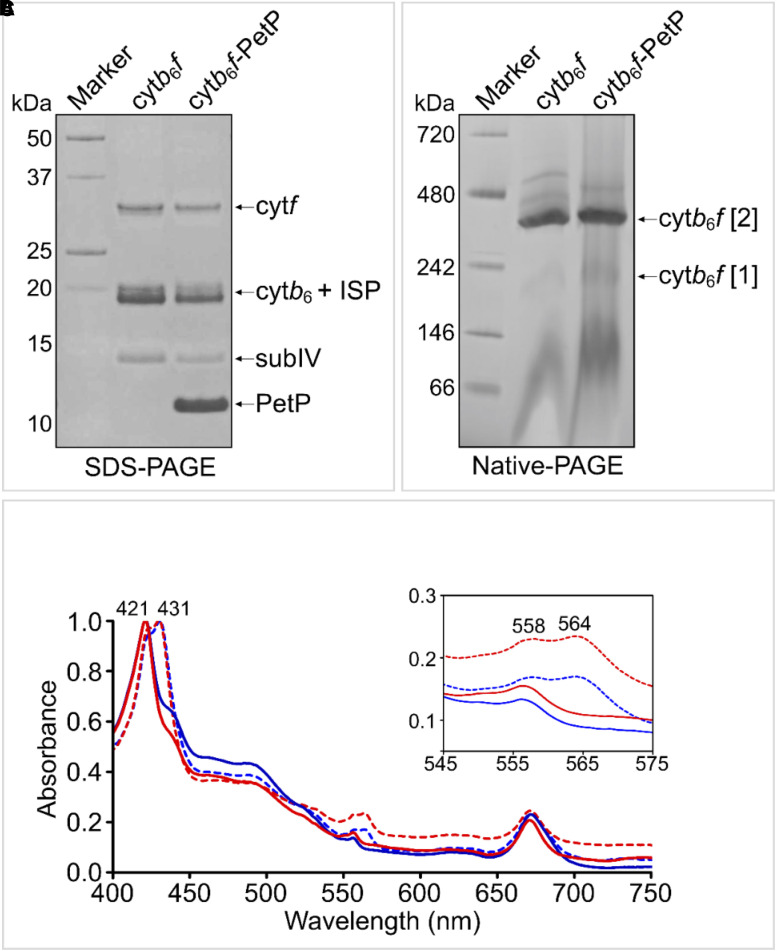
Purification of the *Synechocystis* cyt*b*_6_*f* complex with and without PetP. (**A**) SDS–PAGE separation of purified cyt*b*_6_*f* and the complex reconstituted with PetP (cyt*b*_6_*f*–PetP) followed by staining with Coomassie Brilliant Blue confirmed the presence of cytochrome *f* (cyt*f)*, cytochrome *b*_6_ (cyt*b*_6_), the Rieske ISP, subunit IV (subIV) and PetP. (**B**) Native-PAGE analysis of the same samples showed that the majority of cyt*b*_6_*f* was dimeric (labelled as cyt*b*_6_*f* [2]) with only a small amount of monomeric complex (labelled as cyt*b*_6_*f* [1]). (**C**) Absorbance spectra of cyt*b*_6_*f* (blue) and cyt*b*_6_*f*–PetP (red) after reduction with sodium ascorbate (solid line) and sodium dithionite (dotted line). The inset panel shows a magnified view of the contribution of hemes *b* (564 nm) and *f* (558 nm).

Unlike the filamentous cyanobacteria *Mastigocladus laminosus* and *Nostoc sp*. PCC 7120 previously used to obtain dimeric cyt*b*_6_*f* for structural studies [49,57–64], unicellular *Synechocystis* provides an extremely well-established model system for genetic engineering and site-directed mutagenesis. Previous attempts to purify dimeric cyt*b*_6_*f* from *Synechocystis* were largely unsuccessful due to issues with proteolytic cleavage and monomerization [61]. To circumvent these difficulties, we developed a purification procedure based on solubilization of cyt*b*_6_*f* from membranes of a recombinant strain of *Synechocystis* producing a C-terminally StrepII-tagged PetA (cytochrome *f*) subunit using the mild detergent GDN followed by Strep-Tactin affinity chromatography. The subunit composition, oligomeric state and cofactor content of the purified cyt*b*_6_*f* were verified using SDS- and BN-PAGE and UV/Vis absorption spectroscopy, which showed the four core subunits were present in an intact and dimeric complex, but that PetP was absent (Figure 1A–C).

To reconstitute the cyt*b*_6_*f*–PetP complex, we first over-produced recombinant PetP with an N-terminal His_6_-tag in *E. coli* and purified it by IMAC (Supplementary Figure S3). His_6_-tagged PetP was re-bound to charged Ni^2+^ resin and purified StrepII-tagged cyt*b*_6_*f* was applied to the column; a significant amount of cyt*b*_6_*f* complex was retained on the column after washing, and subsequently co-eluted with PetP (Figure 1A). Control columns lacking PetP did not bind cyt*b*_6_*f,* showing that the complex did not interact non-specifically with the IMAC resin. The subunit composition, oligomeric state and ascorbate-reduced and dithionite-reduced absorption spectra of the cyt*b*_6_*f*–PetP complex were very similar to that of the complex lacking PetP (Figure 1A–C). His-tagged PetP was also able to sequester cyt*b*_6_*f* from solubilized *Synechocystis* thylakoid membranes (Supplementary Figure S4).

### Cryo-EM structures reveal how PetP interacts with cyt*b*_6_*f*

**Figure 2.**
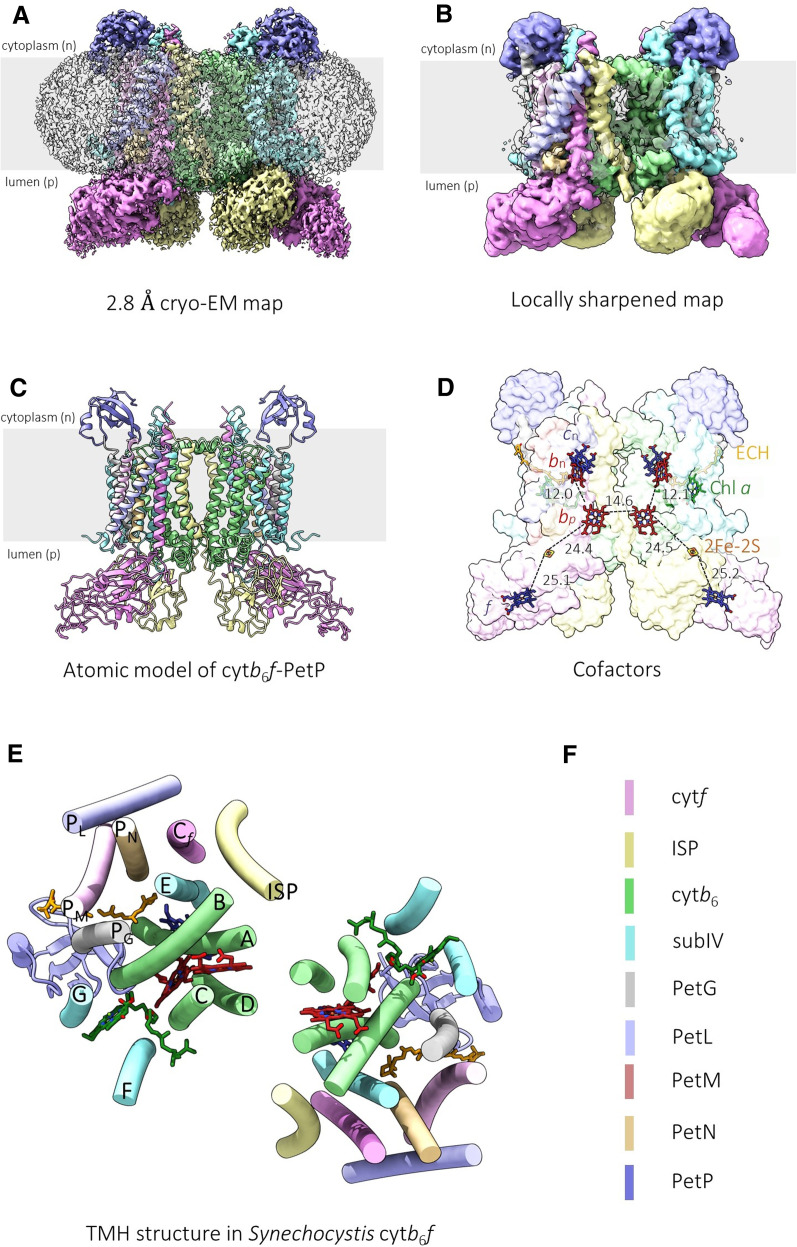
Cryo-EM structure of the *Synechocystis* cyt*b*_6_*f*–PetP complex. (**A**) View of the colour-coded (according to the key in panel F) cyt*b*_6_*f*–PetP density map showing cytochrome *b*_6_ (cyt*b*_6_ green), cytochrome *f* (cyt*f* pink), the Rieske ISP (yellow), subunit IV (subIV cyan), PetG (grey), PetM (salmon), PetN (pale orange), PetL (pale purple) and PetP (dark purple). Detergent and other disordered molecules are shown in semi-transparent light grey. The grey stripe indicates the approximate position of the thylakoid membrane bilayer. (**B**) View of the cyt*b*_6_*f*–PetP density map following local sharpening coloured as in (**A**). (**C**) Modelled subunits of cyt*b*_6_*f* shown in a cartoon representation and coloured as in (**A**). (**D**) Modelled cofactors of cyt*b*_6_*f* showing hemes *b*_n_ and *b*_p_ (both red), hemes *c*_n_ and *f* (both dark blue), the 2Fe-2S cluster (orange Fe and yellow S), Chl *a* (green) and echinenone (orange) in stick representation. The contour levels of the density maps were adjusted to 0.0233 (unsharpened) and 0.0926 (sharpened), respectively. (**E**) The arrangement of TMHs in the cyt*b*_6_*f*–PetP complex.

High-resolution structures of purified *Synechocystis* cyt*b*_6_*f* with (Figure 2) or without (Supplementary Figure S5) PetP were determined by cryo-EM. The global architecture of the *Synechocystis* complex is similar to that of the complexes from other cyanobacteria [57,61], spinach [38] and *Chlamydomonas* [65]. The colour-coded map of the cyt*b*_6_*f*–PetP complex shows the density of the eight subunits in each half of the dimer (Figure 2B,C), which are encircled by a band of disordered density corresponding to the detergent micelle (Figure 2A). At the core of the cyt*b*_6_*f* structure are seven transmembrane helices (TMHs) belonging to cytochrome *b*_6_ (helices A–D) and subunit IV (helices E–G) (Figure 2E). Surrounding these are the single TMHs of PetG, L, M and N and the membrane anchoring helices of the cytochrome *f* and ISP subunits (Figure 2E), with their extrinsic domains projecting from the membrane plane on the p-side of the complex. In the case of the ISP, this domain extends from one monomer to the neighbouring monomer, forming contacts with the neighbouring cytochrome *f* subunit and stabilizing an interlocked dimeric complex. Supplementary Figure S6 shows the density and structural model for each subunit. The positions of the core prosthetic groups (hemes *b*_p_, *b*_n_, *c*_n_, *f* and the 2Fe-2S centre) and the distances between them are shown in Figure 2D, and the corresponding densities in Supplementary Figure S7.

**Figure 3.**
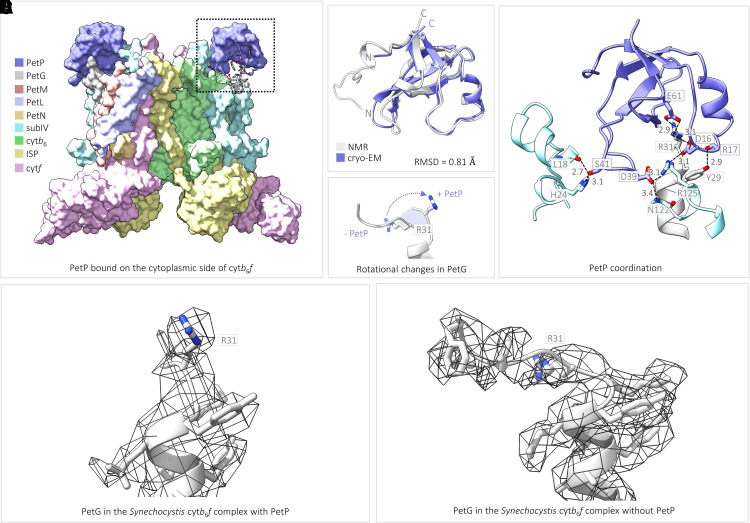
The structure of PetP and its interaction with the cyt*b*_6_*f* complex. (**A**) Surface representation of *Synechocystis* cyt*b*_6_*f* with bound PetP. Subunits are coloured according to the key and residues that form the interaction interface between cyt*b*_6_*f* and PetP are shown in stick representation. (**B**) The modelled structure of PetP in our cryo-EM study (dark purple) superimposed with the NMR structure of PetP (white, PDB ID: 2N5U). (**C**) Zoomed in view of the area highlighted by the black dotted box in (**A**) showing the interaction interface between cyt*b*_6_*f* and PetP. (**D**) Zoomed in view of the conformational changes occurring within the C-terminus of PetG upon binding of PetP. The relative movement of PetG R31 residue to accommodate PetP binding is indicated by the purple dotted arrow. (**E** and **F**) Zoomed in view of the region of the density map corresponding to the C-terminus of PetG in the *Synechocystis* cyt*b*_6_*f* complex with (**E**) and without PetP (**F**). Maps were zoned to within 2.0 Å of the atoms corresponding to PetG in each model; contour levels of the density maps were then adjusted to 0.106 (structure with PetP) and 0.0107 (structure without PetP).

**Figure 4.**
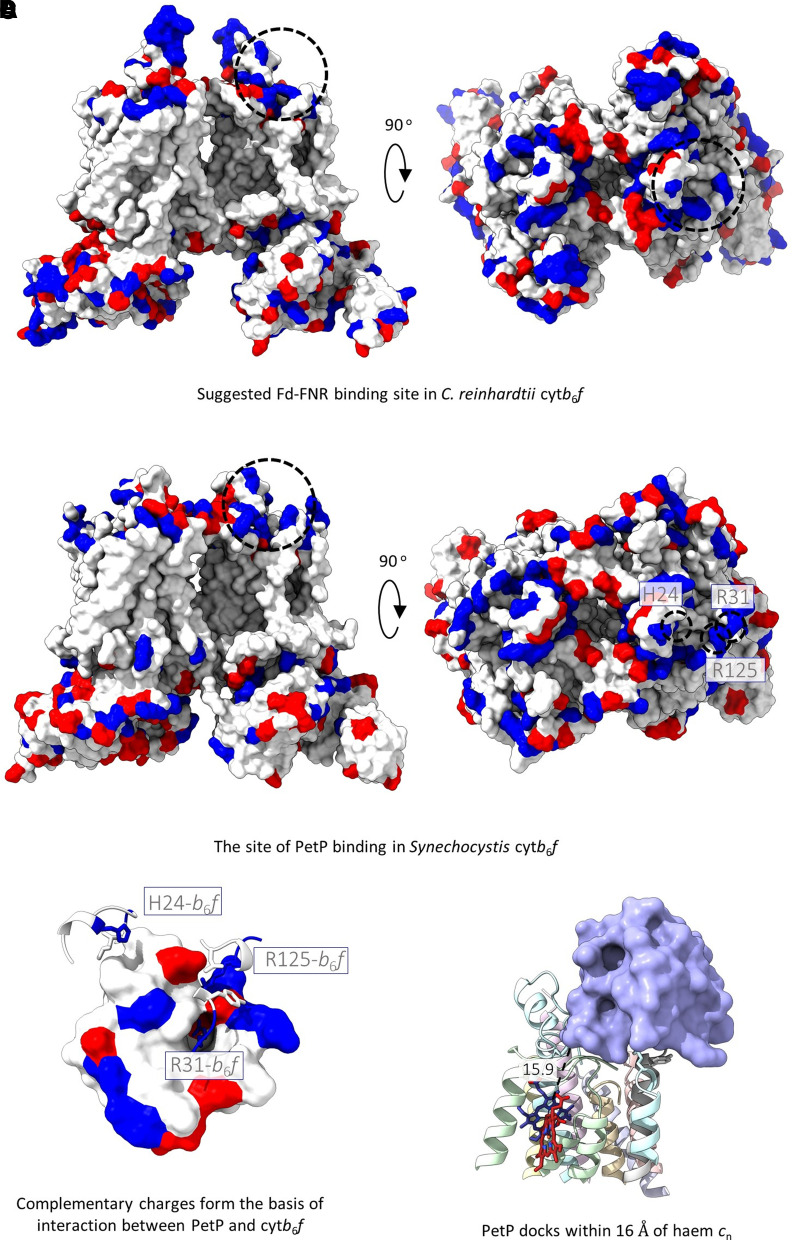
PetP binds to cyt*b*_6_*f* at the proposed Fd-FNR binding site and within 16 Å of heme *c*_n_. (**A**) Surface representation of cyt*b*_6_*f* from *C. reinhardtii* (PDB ID: 1Q90) from the side view (left) and stromal (n-) side of the membrane (right) showing the suggested Fd-FNR binding site [3,66]. Negatively charged residues (Glu, Asp) are coloured red while positively charged residues (Arg, Lys, His) are coloured blue. (**B**) The equivalent surface representation of cyt*b*_6_*f* from *Synechocystis* from the side (left) and cytoplasmic (n-) side of the membrane (right) showing the PetP binding site and specific interacting residues contributing to charge in this region. (**C**) Zoomed in view of the interaction between PetP (surface) and cyt*b*_6_*f* (cartoon) showing how the interaction is mediated by complementary surface charges. (**D**) Zoomed in view of the PetP- cyt*b*_6_*f* interaction showing the proximity of PetP to heme *c*_n_. Subunits and cofactors coloured as in Figure 2.

In the structure of the complex with PetP, additional density was observed on the cytoplasmic side of the complex sitting atop the ‘picket fence’ formed by the PetG, L, M and N subunits and the C-terminus of the cytochrome *b*_6_ subunit (Figure 3A). The structure of this extra protein is consistent with the solution NMR structure of PetP (RMSD 0.81 Å; Figure 3B) [33] and the position is in line with that predicted by crosslinking [30]. PetP forms multiple interactions with the cyt*b*_6_*f* complex including hydrogen bonds between Asp16 (PetP) and Arg125 (subunit IV), Asp16 (PetP) and Arg31 (PetG), Arg17 (PetP) and Tyr29 (PetG), Asp39 (PetP) and both Asn122 (backbone, subunit IV) and Arg125 (subunit IV), and Ser41 (PetP) and both His24 (subunit IV) and Leu18 (subunit IV), as well as a salt bridge between Glu61 (PetP) and Arg31 (PetG) (Figure 3C). Comparative superimposition of the two structures reveals displacement of the C-terminus of the PetG subunit, which moves away from subunit IV to accommodate PetP (Figure 3D–F). PetP binds to the cytoplasmic face of *Synechocystis* cyt*b*_6_*f* at a very similar position to that previously predicted to be the Fd(-FNR) docking site on the stromal surface of the *C. reinhardtii* complex [3,66] (Figure 4A–C), which fits with the suggested role of PetP in modulating the balance between LET and CET in cyanobacteria [30]. The position of PetP binding to the complex and the close proximity of PetP to heme *c*_n_ (the distance between the nearest edge of PetP and the edge of heme *c*_n_ Fe is 15.9 Å, Figure 4D) is noteworthy given that heme *c*_n_ is postulated to act as the conduit for electrons from Fd–FNR in CET [57,65].

**Figure 5.**
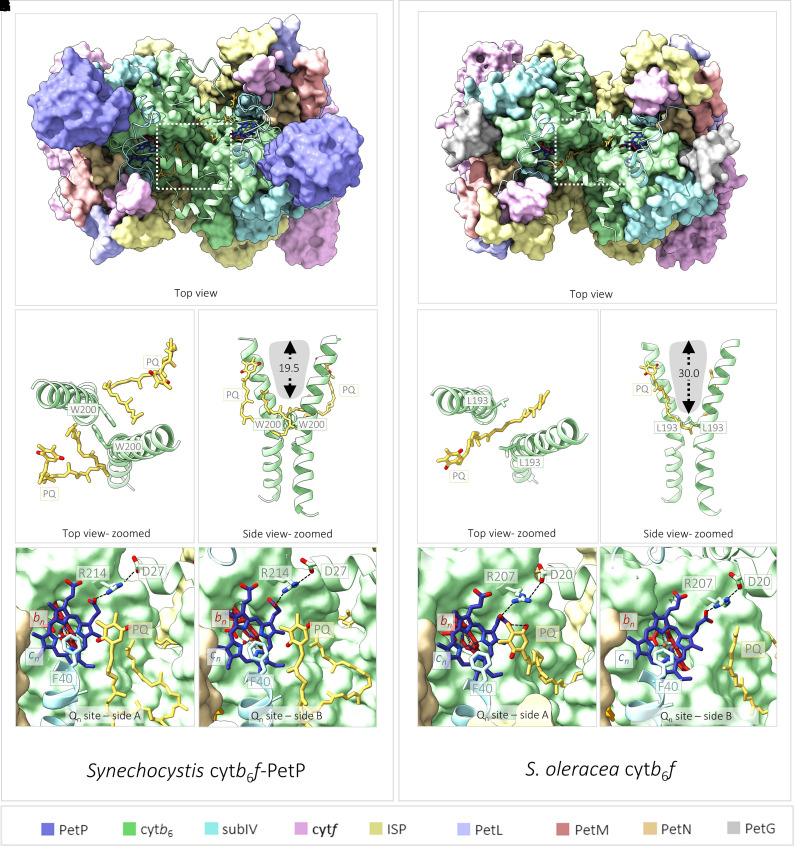
Differences in plastoquinone (PQ) binding at the Q_n_ sites of the *Synechocystis* and *Spinacia oleracea* (spinach) cyt*b*_6_*f* complexes. (**A**) Surface representation of *Synechocystis* cyt*b*_6_*f*–PetP showing that the intermonomer cavity is subdivided by the presence of two Trp residues (W200) and that PQ occupies the Q_n_ sites on either side of the dimer. (**B** and **C**) Zoomed in and simplified view of the area highlighted by the dashed white box in (**A**) viewed from the top (**B**) and side (**C**) showing W200 delimiting the base of the intermonomer cavity and obstructing the passage of substrate between the two halves of the dimer. (**D** and **E**) Magnified views of the *Synechocystis* Q_n_ sites on either side of the complex. (**F**) A surface representation of spinach cyt*b*_6_*f* showing the relative ‘openness’ of the intermonomer cavity in plants compared with cyanobacteria (**A**), allowing a single PQ molecule to span the two sides of the dimer. (**G** and **H**) Zoomed in and simplified view of the area highlighted by the dashed white box in (**F**) viewed from the top (**G**) and side (**H**) showing that the position equivalent to W200 in the cyanobacterial complex is occupied by the substantially smaller Leu residue in the spinach complex, increasing the depth of the cavity. (**I** and **J**) Magnified views of the Q_n_ sites on either side of the spinach complex. Dotted arrows and labels indicate H-bonding distances between atoms. A comparison of the intermonomer cavity sizes between *Synechocystis* and spinach cyt*b*_6_*f* can be obtained by comparing panels (**C**) and (**H**). Grey shaded areas represent protein-free space and black dotted arrows indicate cavity depth. A comparison of substrate binding between the *Synechocystis* and spinach complexes can be obtained by comparing panels (**D**) and (**E**) with panels (**I**) and (**J**); while in the *Synechocystis* complex the Q_n_ sites on either side of the dimeric structure exhibit bound PQ (**D** and **E**), only one Q_n_ site (**I**) is occupied by PQ in the spinach structure while the other (**J**) remains empty. (**K**) Subunit colour key.

### Differences in cofactor binding between the *Synechocystis* and spinach cyt*b*_6_*f* complexes

Previous work suggests that the carotenoid in the *Synechocystis* cyt*b*_6_*f* is all-*trans*-echinenone [67,68] rather than 9-*cis*-β-carotene [61,69–72] or 9-*cis*-α-carotene [73], which are found in other cyt*b*_6_*f* complexes. Our HPLC analysis confirms the enrichment of echinenone in isolated *Synechocystis* cyt*b*_6_*f* compared with solubilized membranes, but also shows the presence of other carotenoids (Supplementary Figure S8G). In our *Synechocystis* cyt*b*_6_*f* structure there is clear density for the ketone group on the end ring of echinenone facing the exterior of the complex, protruding into the thylakoid membrane, which is not present in the spinach structure (Supplementary Figure S8A–D). The position of the carotenoid and its interactions are very similar compared with 9-*cis*-β-carotene in the spinach complex (Supplementary Figure S8E,F), suggesting that it is in the 9-*cis* configuration, consistent with 9-*cis*-carotenes present in other cyt*b*_6_*f* complexes.

The 2.80 Å map of *Synechocystis* cyt*b*_6_*f* with PetP also resolves two native PQ molecules, one bound at each Q_n_ site within the dimer (Figure 5A–E, with corresponding densities in Supplementary Figure S7). This arrangement of Q_n_ site PQ molecules is a key point of difference compared with the spinach cryo-EM structure (Figure 5F–J), where only one Q_n_ site within the dimer is occupied. These differences in PQ binding can be understood by the comparison of the size and shape of the intermonomer cavity, a protein-free region at the heart of the cyt*b*_6_*f* dimer (Figure 5A,F, white boxes). In both the *Synechocystis* and spinach structures the intermonomer cavity is covered at the top by the N-terminal helix of cytochrome *b*_6_ and at the bottom by aromatic residues from the A and D helices of this subunit. The residues that surround the base of the cavity (Phe52 and Phe189 in spinach; Phe59 and Phe196 in *Synechocystis*) are conserved, however, the narrow channel that connects the two sides of the intermonomer cavity is significantly shallower in the *Synechocystis* complex due to the presence of two tryptophan residues (Trp200), which protrude from helix D of the cytochrome *b*_6_ subunit of each monomer (Figure 5B,C). This results in reduction in the cavity depth to ∼19.5 Å in *Synechocystis*, compared with ∼30 Å in spinach (Figure 5G,H). Trp200 is highly conserved in cyanobacterial cyt*b*_6_*f* [49,57,59–64] but in the *Chlamydomonas* [65] and spinach [38] complexes it is substituted for a smaller Leu residue (Supplementary Figure S9). This structural change opens the channel in spinach to allow the entry of the prenyl tail of the Q_n_ bound PQ molecule, potentially enabling PQ bound at the Q_n_ site of one monomer to obstruct the Q_n_ site of the opposite monomer, such that the binding of substrate at either of the Q_n_ sites is a mutually exclusive event (Figure 5G–J). Interestingly, the positioning of the prenyl tail of the Q_n_ bound PQ molecules in the *Synechocystis* complex is quite different relative to the spinach complex, with each prenyl tail confined to a single side of the cyt*b*_6_*f* dimer by the presence of the Trp200 sidechains (Figure 5B–E).

Slight differences between the *Synechocystis* and spinach complexes are also seen in the mode of binding of the 1,4-benzoquinone head group of the PQ molecule to the heme *c*_n_. In spinach, binding of the PQ substrate induces a conformational change in the heme *c*_n_ propionate group, bending the propionate group away from its interaction with Arg207 (Arg214 in *Synechocystis*) and forming a H-bond with the carbonyl group of the 1,4-benzoquinone ring of the PQ (Figure 5I). In *Synechocystis*, the propionate group of heme *c*_n_ adopts a conformation similar to that observed in the empty Q_n_ site in the spinach cyt*b*_6_*f* dimer (Figure 5J), forming a hydrogen bond to Arg214 in both halves of the dimer (Figure 5D,E). We therefore suggest that the *Synechocystis* structure may capture the approach of the PQ molecule to the Q_n_ site prior to tight binding (Figure 5D,E). It was previously suggested that the Phe40 residue of subunit IV might be displaced from its position capping the heme *c*_n_ tetrapyrrole ring by PQ binding [74], however we observe little change in its position (Figure 5D,E,I,J).

In addition to the observed density at the Q_n_ site, we also observe some density at the Q_p_ sites in the *Synechocystis* structure (Supplementary Figure S10A,B). In contrast with the clear densities assigned to PQ at each Q_n_ site, the densities at the Q_p_ sites are much weaker and cannot be unambiguously assigned as PQ. However, superimposition of these densities onto the *M. laminosus* structure with bound tridecylstigmatellin (PDB ID: 4H13) [62] is consistent with PQH_2_ bound in the 2Fe-2S proximal lobe of the Q_p_ site (Supplementary Figure S10C,D).

## Discussion

The central role of the cyt*b*_6_*f* complex in the LET and CET chains necessitates its careful regulation, and auxiliary proteins have evolved to modulate these functions. Yet, due to the transient and weak nature of their binding, high-resolution structural details of these interactions have so far remained elusive. Here, we reconstituted the cyt*b*_6_*f*–PetP complex from the model cyanobacterium *Synechocystis in vitro* and determined its structure compared with the cyt*b*_6_*f* complex without PetP, providing new insights into how this peripheral subunit interacts with cyt*b*_6_*f*. The high-resolution structures also reveal details of the mode of binding of the PQ substrate to the Q_n_ site and differences between the cyanobacterial and plant/algal complexes.

PetP binds to the cytoplasmic side of the cyt*b*_6_*f* complex. Our column pulldowns with PetP did not reveal any evidence for interacting partners beyond cyt*b*_6_*f*, suggesting that PetP is not involved in mediating supercomplex formation with other photosynthetic complexes, as proposed previously [30]; the PetM subunit has recently been suggested to fulfil this function in *Synechococcus elongatus* [75]. We suggest that the proposed function of PetP in mediating LET and CET [30] is explained by its binding position at the crucial C-terminus of the cytochrome *b*_6_ subunit that coordinates heme *c*_n_. Notably, crosslinking data suggest that both the kinase Stt7 and the algal-specific PetO subunit also bind to a similar region of cytochrome *b*_6_ and subunit IV [23,76]. Previously it has been shown that perturbation of the C-terminus of the cytochrome *b*_6_ subunit affects the binding affinity and properties of heme *c*_n_ [76,77], while phosphorylation of the C-terminus of subunit IV (Thr4) by Stt7 appears to modulate CET in *Chlamydomonas* [78]. Given the suggested role of heme *c*_n_ as the conduit for electrons from Fd to the PQ pool during CET [57,65], and the phenotype of the Δ*petP* mutant, which shows diminished LET relative to CET [30], we suggest that PetP competes with Fd–FNR for binding on the cytoplasmic surface of cyt*b*_6_*f*. In such a scenario, PetP has a role in sequestering cyt*b*_6_*f* complexes exclusively for LET; when PetP is absent the balance of LET compared with CET is altered as more complexes can bind Fd–FNR. The lack of PetP in plants and green algae may be explained by the presence of thylakoid membrane stacking, which is known to play a part in regulating the LET/CET balance [79,80]. The segregation of part of the cyt*b*_6_*f* population within the thylakoid stacks [81] would protect it from interaction with Fd-FNR, removing the necessity for PetP (Supplementary Figure S11). PetP homologues are also found in red algae [30]; like cyanobacteria, red algae contain phycobilisomes and have unstacked thylakoid membranes, which may necessitate PetP-mediated regulation of LET and CET. The dual presence of PetP and phycobilisomes in cyanobacteria and red algae led Rexroth et al. [30] to suggest a link between PetP and regulation of state transitions, although recent evidence suggests cyt*b*_6_*f* is not involved in this process in cyanobacteria [11].

Unlike the 1,4-benzoquinone ring headgroup, the binding orientation of the prenyl tail of the PQ molecule at Q_n_ is quite different in spinach and *Synechocystis*. In the *Synechocystis* cyt*b*_6_*f*, the prenyl tail of the PQ molecule bound at Q_n_ cannot straddle the intermonomer cavity due to the presence of two bulky tryptophan residues. This prevents the extended prenyl tail of PQ bound to one Q_n_ site from obstructing the binding of a second PQ to the opposing Q_n_ site, as seen in spinach [38]. It is possible that the Q_n_ blocking mechanism observed in spinach may have evolved in plants and green algae to facilitate the rapid production of PQH_2_ under oxidizing conditions (e.g. low light). Blocking one Q_n_ site could ensure turnover of the low potential chain and production of PQH_2_ in two rather than three PQH_2_-oxidizing turnovers, either via a second electron transfer from the Q_p_ site of the same monomer, or by inter-monomer ET via the heme *b*_p_ cofactors in the low potential chain in the neighbouring monomer, as previously suggested [64,82]. In this scenario, competition between the two Q_n_ sites is decreased; therefore, the lifetime of unpaired electrons, which may give rise to reactive oxygen species (ROS), is minimized. In contrast, co-localisation of quinone-reducing respiratory complexes such as succinate dehydrogenase and NDH-1 in cyanobacterial thylakoid membranes may negate the need for such adaptations by keeping the redox poise more reducing, even under low light conditions. This would keep the flux of quinol into the cyt*b*_6_*f* complex sufficiently high to allow both monomers to operate independently because semiquinone species will not be sufficiently long-lived to generate ROS.

In summary, alongside recent high-resolution structures of *Synechocystis* PSI [83,84] and PSII [85], the 2.8 Å structure of *cytb*_6_*f* completes the set of the three core photosynthetic electron transfer complexes from this model mesophilic species. The simple method of purification described here now allows us to contemplate detailed structure-based mutagenesis to settle unresolved questions regarding the function and regulation of this crucial complex.

## Data Availability

The cryo-EM density maps have been deposited in the Worldwide Protein Data Bank (wwPDB) under accession code EMD-14224 (+PetP) and EMD-15017 (−PetP) and the coordinates have been deposited in the Protein Data Bank (PDB) under accession numbers 7R0W (+PetP) and 7ZXY (−PetP).

## Abbreviations

*cat*: chloramphenicol acetyl transferase
CET: cyclic electron transfer
cryo-EM: cryogenic electron microscopy
cyt*b*_6_*f*: cytochrome *b*_6_*f*
Fd: ferredoxin
FNR: ferredoxin-NADP^+^ reductase
GDN: glyco-diosgenin
IMAC: immobilized metal affinity chromatography
ISP: iron-sulfur protein
LET: linear electron transport
MWCO: molecular weight cut-off
NDH-1: NADH dehydrogenase-like complex 1
NMR: nuclear magnetic resonance
Pc: plastocyanin
PGR: Proton Gradient Regulation
PQ: plastoquinone
PQH_2_: plastoquinol
PSI: photosystem I
PSII: photosystem II
ROS: reactive oxygen species
RP-HPLC: reversed-phase high-performance liquid chromatography
WT: wild type
