# Supplementary material for: Cryo-EM structures of the Synechocystis sp. PCC 6803 cytochrome b6f complex with and without the regulatory PetP subunit
Source: Biochem J. 2022 Jul 15;479(13):1487–503. doi: 10.1042/BCJ20220124 (PMC9342900; doi:10.1042/BCJ20220124)
Supplement: Supplementary Material [file BCJ-479-1487-s1.pdf]

Supplementary information for:

Cryo-EM structures of the *Synechocystis* sp. PCC 6803 cytochrome b_6f complex with and without the regulatory PetP subunit

Matthew S. Proctor[1§], Lorna A. Malone[1,2§], David A. Farmer[2§], David J.K. Swainsbury[1,3], Frederick R. Hawkings[1,2], Federica Pastorelli[1], Thomas Z. Emrich-Mills[1], C. Alistair Siebert[2], C. Neil Hunter[1], Matthew P. Johnson[1]* and Andrew Hitchcock[1]*

[1]Plants, Photosynthesis and Soil, School of Biosciences, University of Sheffield, Sheffield S10 2TN, UK.

[2]Electron Bio-imaging Centre, Diamond Light Source, Didcot, UK.

[3]School of Biological Sciences, University of East Anglia, Norwich NR4 7TJ, UK.

[§]These authors contributed equally

*Corresponding authors: a.hitchcock@sheffield.ac.uk and matt.johnson@sheffield.ac.uk

Author ORCID IDs: 0000-0002-1484-850X (MSP); 0000-0002-7625-7545 (LAM); 0000-0001-5331-3551 (DAF); 0000-0002-0754-0363 (DJKS); 0000-0001-6741-920X (FRH); 0000-0001-5322-4479 (FP); 0000-0002-4440-626X (TZE-M); 0000-0002-8126-1979(CAS); 0000-0003-2533-9783 (CNH); 0000-0002-1663-0205 (MPJ); 0000-0001-6572-434X (AH).

Table S1. Primers used in this study.

Name	Sequence (5' to 3')	Details
cat-F	TACCGGGAAGCCCTGGGCC	Primers for amplification of *cat* gene
cat-R	TTACGCCCCGCCCTGCCAC	
petA-ds-F	CGATGAGTGGCAGGGCGGGGCGTAATTCATTTCTCCACCGGATTATCCC	*petA* downstream homology region amplification primers with overlap with *cat* gene
petA-ds-R	ATGTAACAAGCTTGTGTTTTGGGCTTCCCGGGCCGCCTGC	
petA-SII-F	TTAGCAGGATCCAGCAGTATCAGG	Primers for OLE-PCR amplification of *petA*-StrepII linear DNA construct
petA-SII-R	ATGTAACAAGCTTGTGTTTTGGGCTTCC	
petA-screen-F	CATGGAAAATGTGGTCATTGTTGG	*petA* locus screening primers
petA-screen-R	CATCGGAAAATTCGTTGTCTGG	

Table S2. Cytb_6f cryo-EM data acquisition, model refinement and validation statistics

**Parameters**	**(EMD-15017, PDB 7ZXY)**
**Data collection**	
Nominal Magnification	81,000 X
Accelerating Voltage (kV)	300
Electron dose rate ($e^-/Å^2/s$)	15.0
Exposure time (s)	3.0
Number of fractions	45
Defocus range (-µm)	-1.2 to -2.5
Pixel size (Å)	0.53
Symmetry imposed	C1
Initial particle images (no.)	4,032,212
Final particle images (no.)	413,442
Map resolution (Å) (global)	3.15
FSC threshold	0.143
Map resolution range (Å)	~2.95 – 5.32
**Refinement**	
Initial model used	RELION *de novo* model
FSC threshold	0.143
Map sharpening *B* factor ($Å^2$)	Estimated automatically using RELION*
Model composition	
Non-hydrogen atoms	15,609
Protein residues	1,913
Waters	0
Ligands	23
B factors ($Å^2$)	
Protein	RELION auto-estimated
Ligand	RELION auto-estimated
RMS deviations (PHENIX)	
Bond lengths (Å)	0.004
Bond angles (°)	0.652
**Validation**	
Molprobity score	1.77
Clashscore	10.58
Poor rotamers (%)	0.00

Ramachandran plot

Favoured (%)	96.53
Allowed (%)	3.47
Disallowed (%)	0.00

Table S3. Cytb_6f-PetP cryo-EM data acquisition, model refinement and validation statistics

**Parameters**	**(EMD-14224, PDB 7R0W)**
**Data collection**	
Nominal Magnification	81,000 X
Accelerating Voltage (kV)	300
Electron dose rate (e^-/Å^2/s)	14.8
Exposure time (s)	3.5
Number of fractions	50
Defocus range (-µm)	-1.2 to -2.4
Pixel size (Å)	1.06 (0.53 at super-resolution, pre-binned with EPU)
Symmetry imposed	C1
Initial particle images (no.)	1,169,445
Final particle images (no.)	152,860
Map resolution (Å) (global)	2.82
FSC threshold	0.143
Map resolution range (Å)	~2.5 – 5.5
**Refinement**	
Initial model used	RELION *de novo* model
FSC threshold	0.143
Map sharpening *B* factor (Å^2)	Estimated automatically using RELION*
Model composition	
Non-hydrogen atoms	16,830
Protein residues	2,040
Waters	2
Ligands	27
B factors (Å^2)	
Protein	RELION auto-estimated
Ligand	RELION auto-estimated
RMS deviations (PHENIX)	
Bond lengths (Å)	0.005
Bond angles (°)	0.947
**Validation**	
Molprobity score	1.78
Clashscore	11.64
Poor rotamers (%)	0.84

Ramachandran plot	
Favoured (%)	96.79
Allowed (%)	3.21
Disallowed (%)	0.00

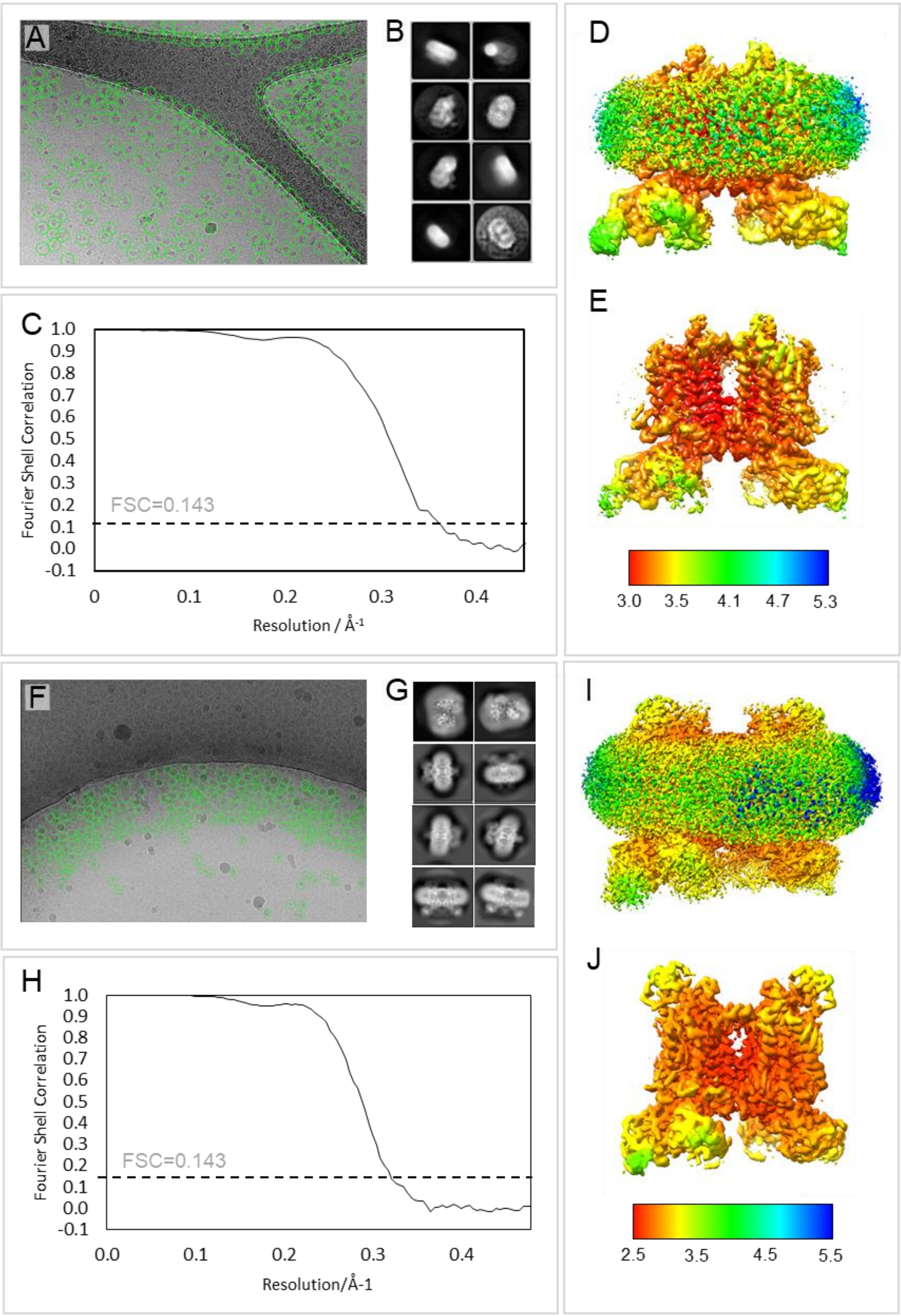

Figure S1. Cryo-EM micrographs of the *Synechocystis* cytb_6f and cytb_6f-PetP complexes and calculation of the cryo-EM map global and local resolution. (A) A representative micrograph showing autopicked coordinates. (B) Selection of 2D classes following two successive rounds of 2D classification. (C) 'Gold-standard' refinement was used for estimation of the final map resolution (solid black line). The global resolution of 3.15 Å was calculated using a FSC cut-

off at 0.143. (D, E) A C1 density map of the cytb_6f complex both with (D) and without (E) the detergent shell. The map is coloured according to local resolution estimated by RELION and viewed from within the plane of the membrane. The colour key below shows the local structural resolution in angstroms (Å). (F-J) the same as in (A-E) but for the cytb_6f-PetP dataset (global resolution of 2.80 Å estimated by the FSC at 0.143 in (H)).

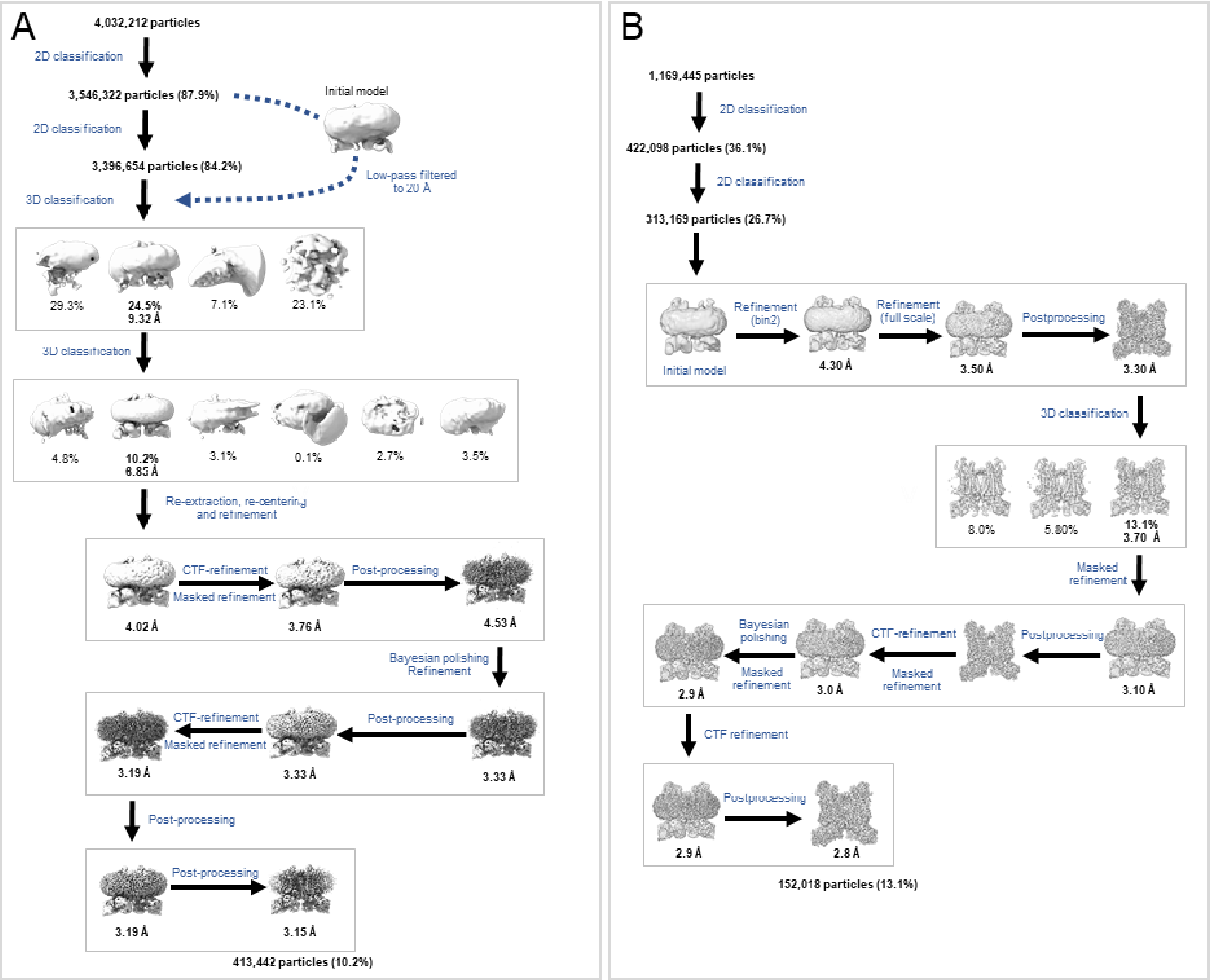

Figure S2. Flowcharts detailing the processing of the *Synechocystis* cytb_6f and cytb_6f-PetP cryo-EM maps in RELION. (A) 18,151 cryo-EM movies were recorded of the cytb_6f complex, from which 4,032,212 particles were auto-picked for reference-free 2D classification. The final density map was calculated from 413,442 particles. The average resolution in angstroms (Å) and number of particles (%) at each stage is indicated with the final global resolution for the entire cytb_6f complex from *Synechocystis* estimated to be ~ 3.15 Å. (B) 20,133 cryo-EM movies were recorded of the cytb_6f-PetP complex, from which 1,169,445 particles were auto-picked for reference-free 2D classification. The final density map was calculated from 152,018 particles. The average resolution in angstroms (Å) and number of particles (%) at each stage is indicated with the final global resolution for the entire cytb_6f-PetP complex estimated to be ~ 2.80 Å.

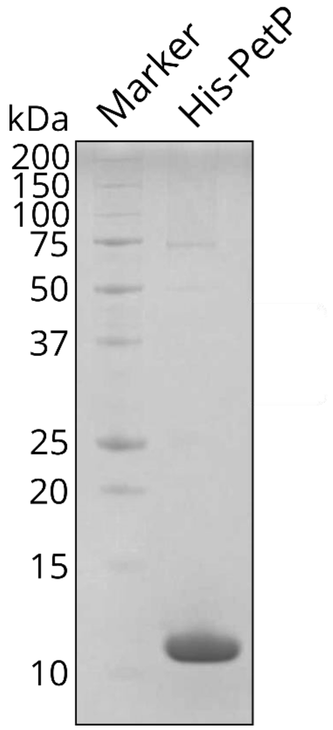

Figure S3. SDS-PAGE analysis of purified His_6-PetP used in reconstitutions with purified cyt*b_6f*. His_6-tagged PetP was produced in *E. coli* and purified by IMAC.

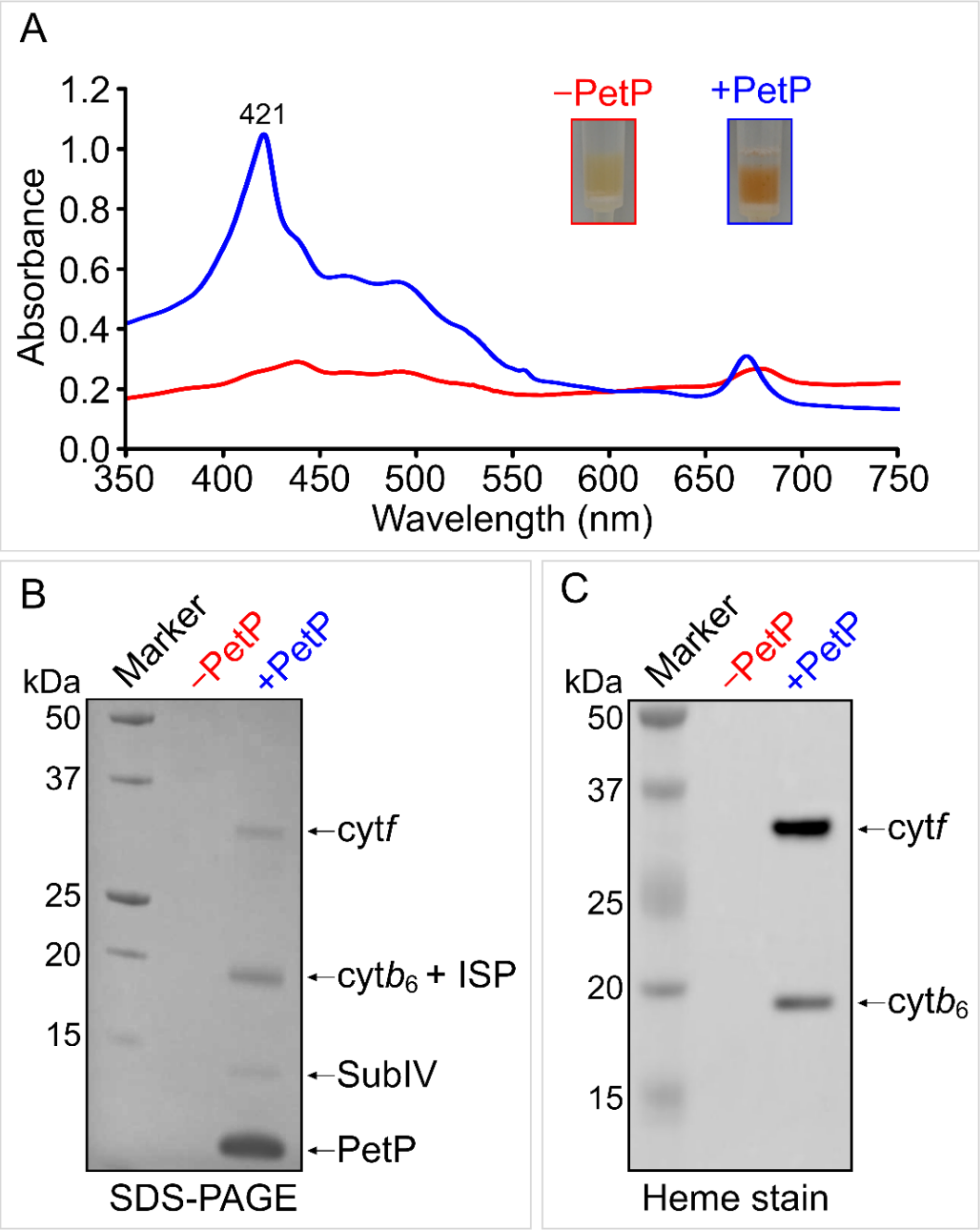

Figure S4. Column-immobilised His$_6$-PetP pulls down cytb_6f from detergent-solubilised *Synechocystis* membranes. Membranes were solubilised with 1.5% (w/v) GDN and applied to Ni^{2+}-NTA agarose columns with bound His-tagged PetP (+PetP, blue traces/labels). After washing, PetP was eluted with L-histidine and the eluates were analysed by absorbance spectroscopy (A), SDS-PAGE (B) and heme staining (C), which showed that cytb_6f co-immunoprecipitated with PetP. Control columns lacking PetP (-PetP, red traces/labels) did not retain cytb_6f.

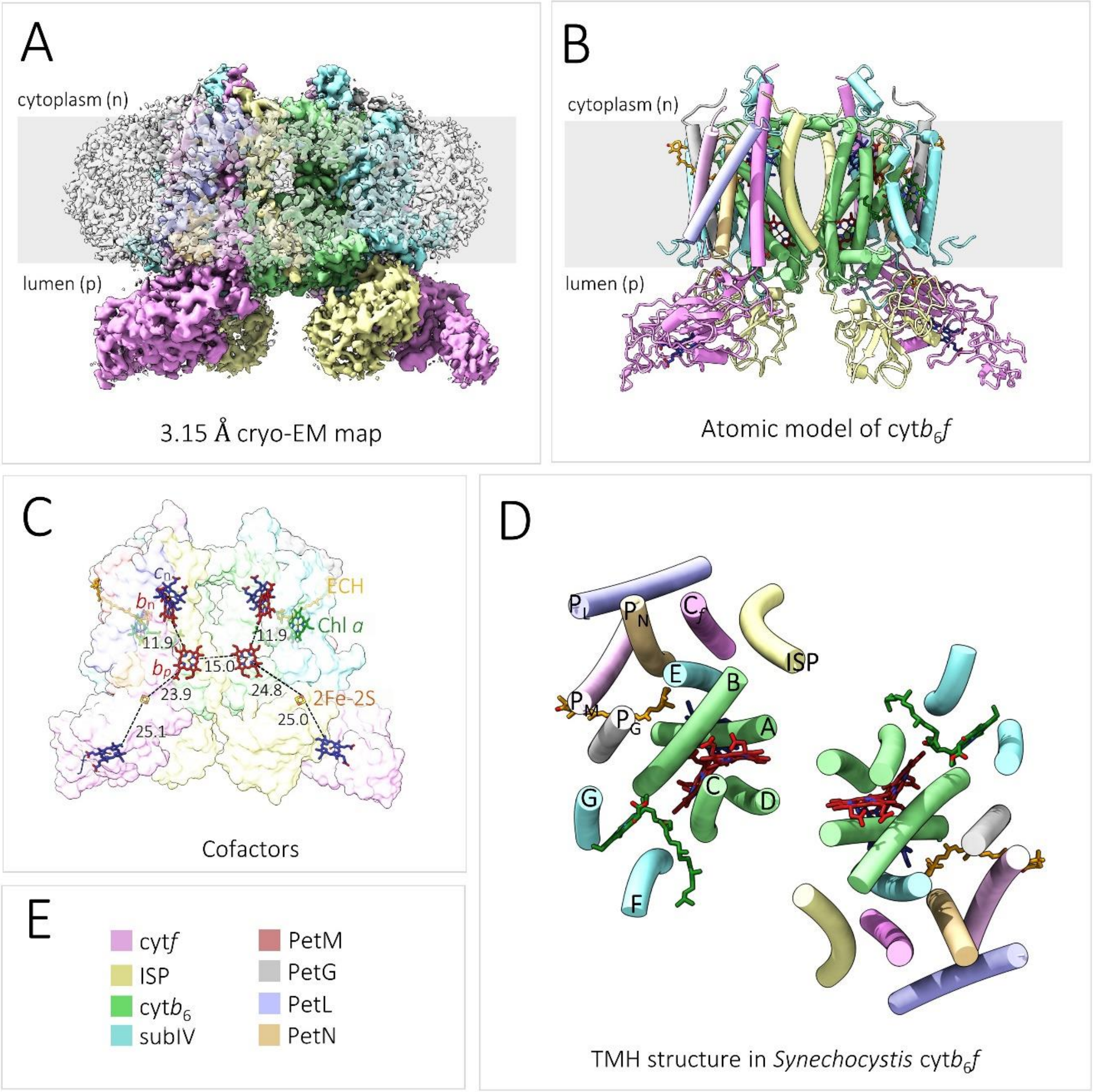

Figure S5. Cryo-EM structure of the *Synechocystis* cyt*b₆f* complex. (A) The colour-coded cytb_6f density map showing cytochrome b_6 (cytb_6 green), cytochrome *f* (cyt*f* pink), ISP (yellow), subIV (cyan), PetG (grey), PetM (salmon), PetN (pale orange) and PetL (pale purple). Detergent and other disordered molecules are shown in semi-transparent light grey. The contour level of the density map was adjusted to 0.0221. (B) The modelled protein structure of *Synechocystis* cytb_6f. The grey stripe indicates the probable position of the thylakoid membrane bilayer. (C) Modelled cofactors of cytb_6f showing heme b_n (firebrick red), heme b_p (firebrick red), heme c_n (dark blue), heme *f* (dark blue), the 2Fe-2S (orange Fe and yellow S), chlorophyll *a* (green) and echinenone (orange) in stick representation. (D) The arrangement of TMHs in the *Synechocystis* cytb_6f complex. (E) Subunit colour key.

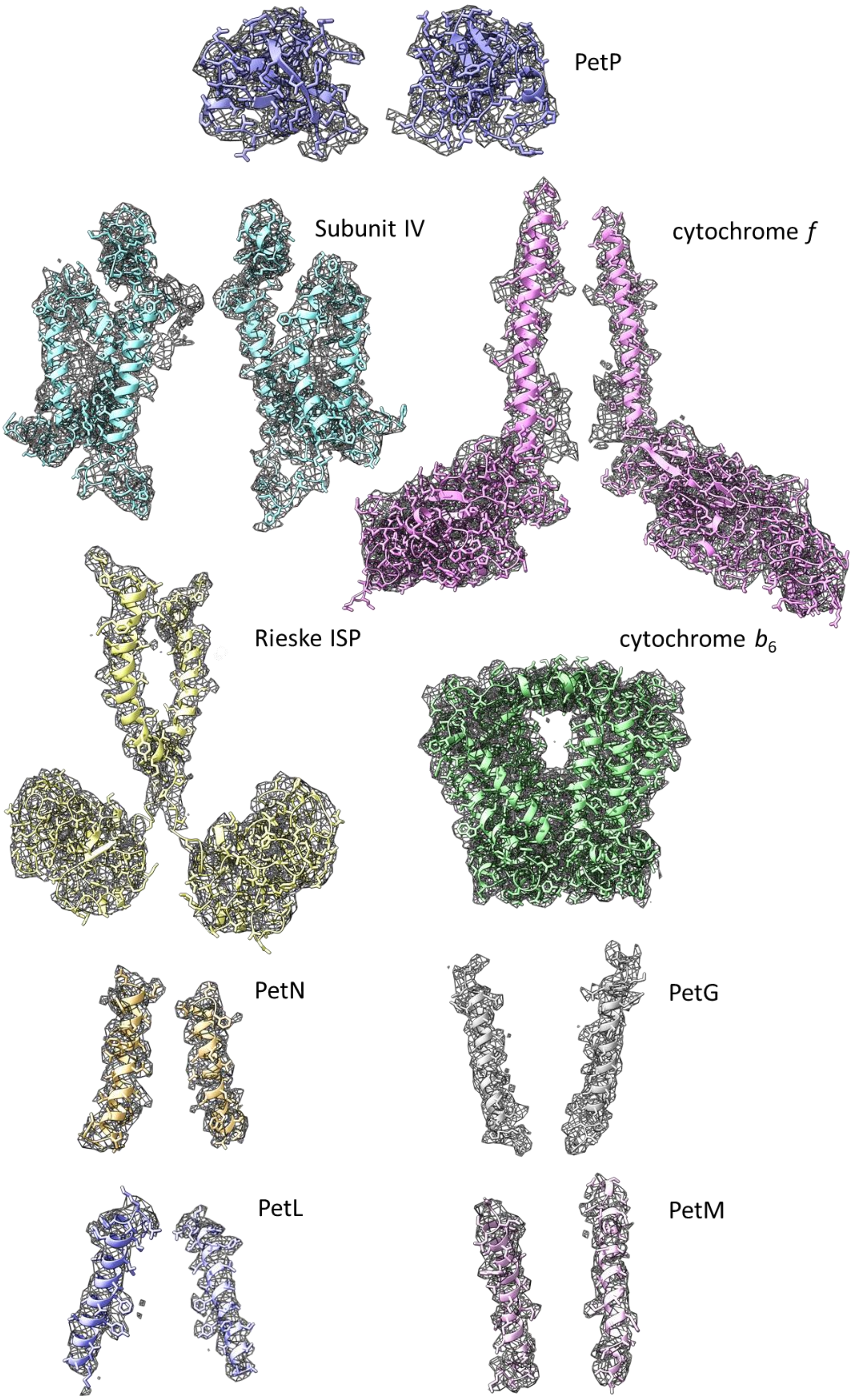

Figure S6. Cryo-EM densities and structural models of polypeptides in the *Synechocystis* cytb_6f-PetP complex. The colour code is the same as in Figure 2. The contour levels of the density maps were adjusted to 0.0926.

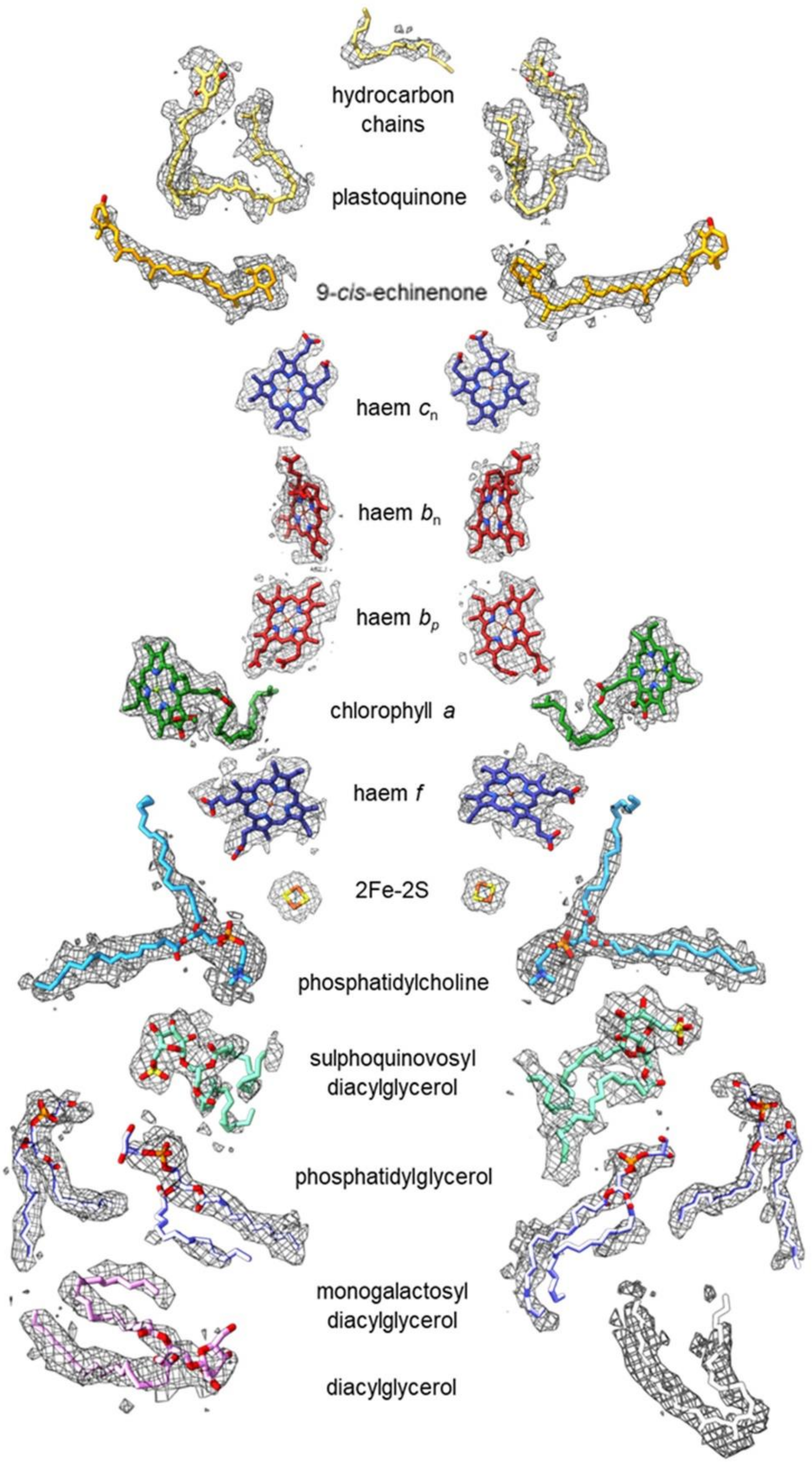

Figure S7. Cryo-EM densities and structural models of prosthetic groups, lipids and plastoquinone molecules in the cyt*b*$_6$*f*-PetP complex. Molecules are colour coded with plastoquinones and hydrocarbon chains (yellow), 9-*cis*-echinenone (orange), hemes *f* and c_n (dark blue), hemes b_p and b_n (red), chlorophyll *a* (dark green), 2Fe-2S (orange/yellow), phosphatidylcholine (sky blue), sulphoquinovosyldiacylglycerol (mint green), phosphatidylglycerol (light purple), monoglalactosyldiacylglycerol (light pink) and diacylglycerol (white). The contour levels of the density maps were adjusted to 0.109.

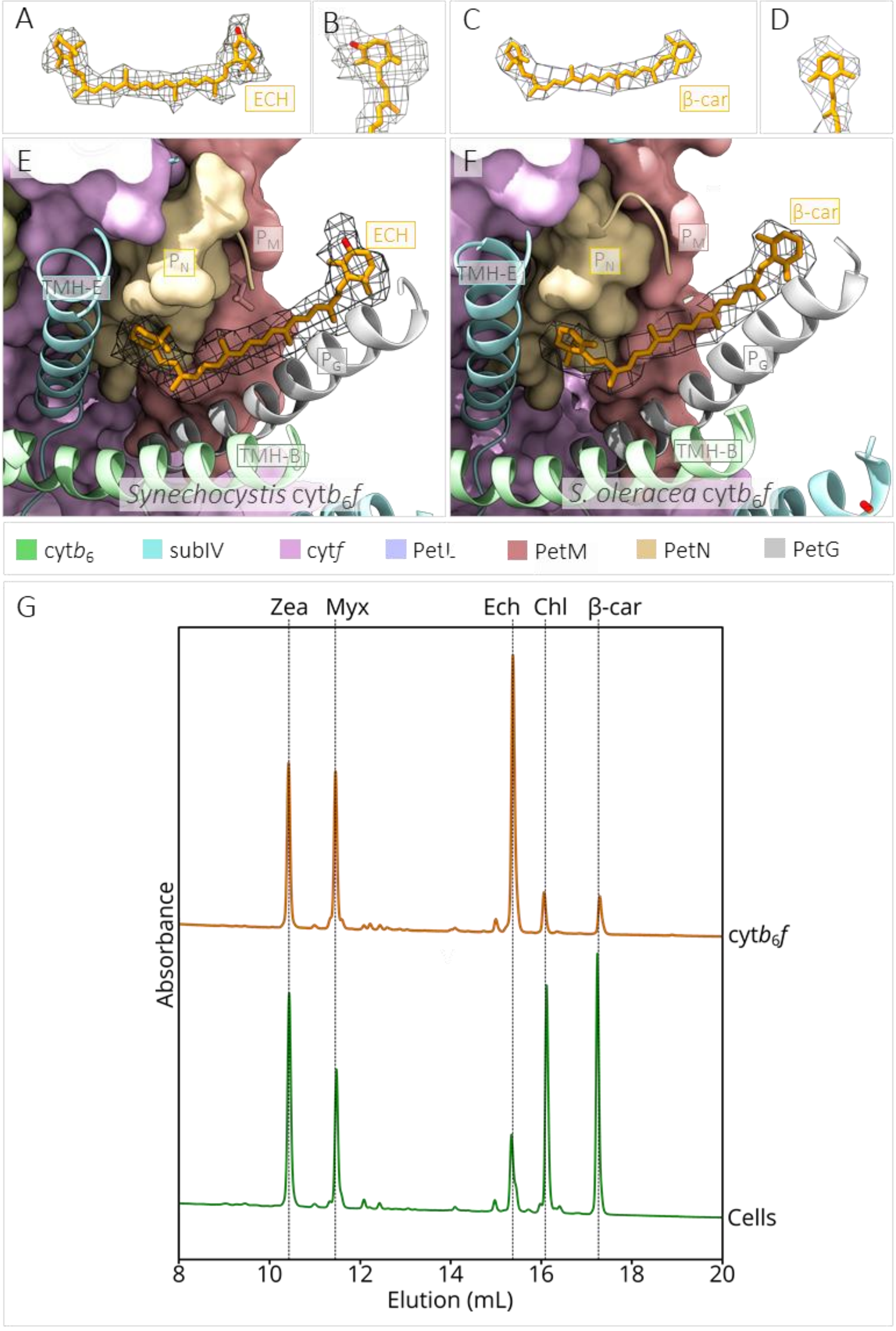

Figure S8. **The carotenoids of cyt*b*$_6$*f*.** (A-D) A comparison of the cryo-EM densities of the region corresponding to the carotenoid molecule in the *Synechocystis* cyt*b*$_6$*f* (A, B) and *S. oleracea* cyt*b*$_6$*f* (C, D) complexes. Both 9-*cis* echinenone (ECH) and 9-*cis* β-carotene (β-car) are coloured orange with the =O of the ketone in echinenone coloured red. For clarity, maps were zoned to within 2.0 Å of the atoms corresponding to either carotenoid in each model; contour levels of the density maps were then adjusted to 0.0137 (*Synechocystis*) and 0.00701 (*S. oleracea*) respectively. (E, F) Surface representation of cryo-EM structures of ECH and β-car bound within the *Synechocystis* (E) and *S. oleracea* (F) cyt*b*$_6$*f* structures, respectively. Protein subunits are colour and letter coded as in Figure 2. (G) Reversed-phase high-performance liquid chromatography profile of pigments extracted from *Synechocystis* cyt*b*$_6$*f* (orange trace) and whole cells (green trace) monitoring at 450 nm. Zeaxanthin (Zea), myxoxanthophyll (Myx), echinenone (ECH), chlorophyll (Chl) and β-carotene (β-car) pigments were identified by their absorbance spectra (not shown).

```
Synechocystis    MRGSESVGQATLTRFYSLHTFVLPWAIAVLLLLHFLMIRKQGISGPL 222
M. laminosus     LRGGSSVGQATLTRYYSAHTFVLPWLIAVFMLLHFLMIRKQGISGPL 215
Nostoc           LRGGSSVGQATLTRYYSAHTFVLPWLIAVFMLFHFLMIRKQGISGPL 215
C. reinhardtii   LRGGVGVGQATLTRFYSLHTFVLPLLTAVFMLMHFLMIRKQGISGPL 215
S. oleracea      LRGSASVGQSTLTRFYSLHTFVLPLLTAVFMLMHFLMIRKQGISGPL 215
A. thaliana      LRGSASVGQSTLTRFYSLHTFVLPLLTAVFMLMHFLMIRKQGISGPL 215
                 :**. .***:****:** ******   **::*:**************
```

Figure S9. Sequence alignment of cytochrome *b*$_6$ subunits from cyanobacteria, algae and plants. Cytochrome *b*$_6$ subunits from *Synechocystis* sp. PCC 6803 (*Synechocystis*), *Mastigocladus laminosus* (*M. laminosus*), *Nostoc* sp. PCC 7120 (*Nostoc*), *Chlamydomonas reinhardtii* (*C. reinhardtii*), *Spinacia oleracea* (*S. oleracea*) and *Arabidopsis thaliana* (*A. thaliana*) were aligned using Clustal Omega. Trp (*Synechocystis*, *M. laminosus* and *Nostoc*) and Leu (*C. reinhardtii*, *S. oleracea*, *A. thaliana*) residues are highlighted in bold red text. The key denotes conserved residues (*) and conservative (:), semi-conservative (.) and non-conserved ( ) substitutions.

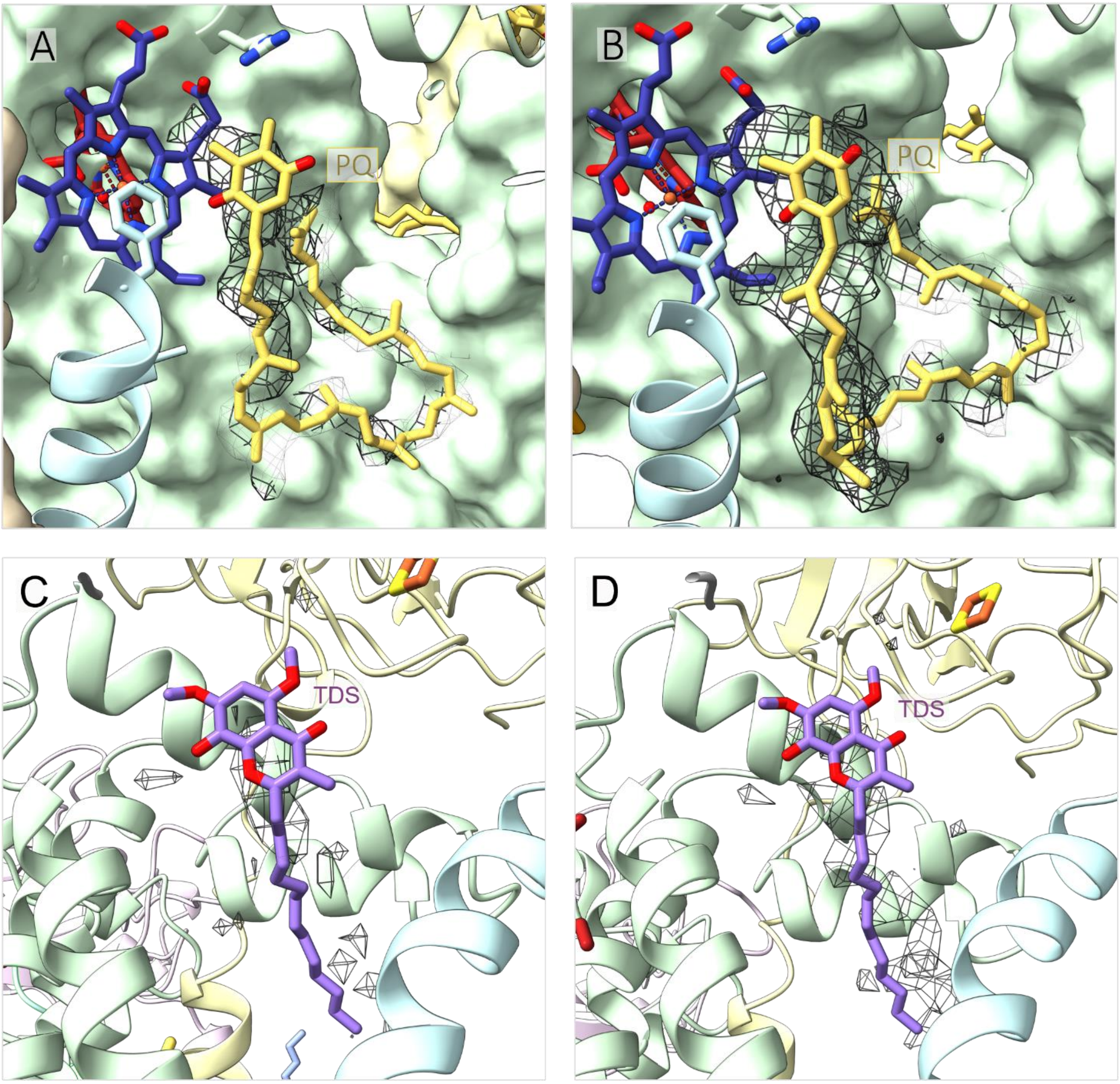

Figure S10. Density at the cyt*b*$_6$*f*-PetP Q$_p$ sites. (A, B) Density at the Q$_n$ site assigned as PQ on either side of the dimeric *Synechocystis* cyt*b*$_6$*f*-PetP complex. (C, D) Weak density within the Q$_p$ site overlays well with superimposed tridecylstigmatellin (TDS) bound in the 2Fe-2S proximal position on either side of the dimeric complex. Protein subunits are coloured as in Figure 2. A protein-free map was created in Coot and used to identify ligand positions. Here the region of the protein-free map corresponding to the Q$_p$ site is displayed and superimposed on the protein map. For clarity, the map is restricted to include only regions within 3.0 Å of the superimposed TDS molecule. Contour levels of the density maps were adjusted to 0.242 for the Q$_p$ site and 0.166 for the Q$_n$ site.

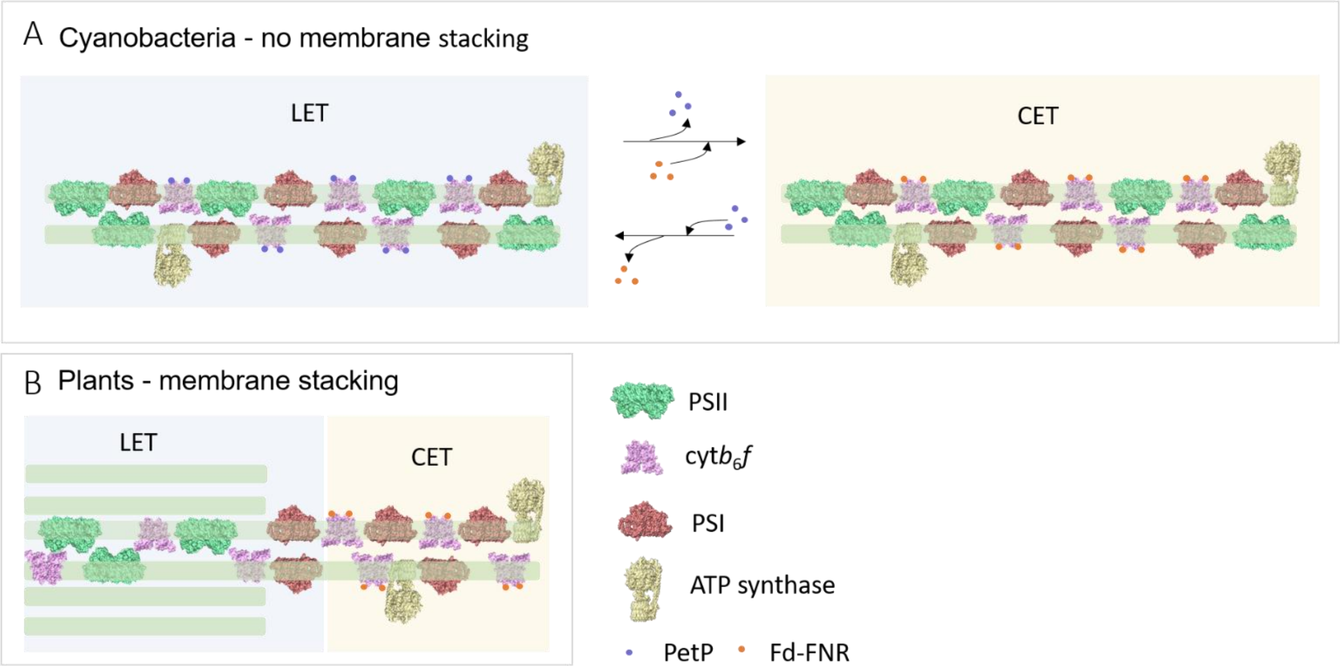

Figure S11. Schematic model of LET/CET regulation at cytb_6f in cyanobacteria versus plants. (A) In cyanobacteria thylakoid membrane stacking is absent and the proportion of cytb_6f complexes involved in LET versus CET is regulated by the relative proportions binding PetP versus Fd-FNR. (B) In contrast, in plants thylakoids form tight grana stacks whose appressions prevent access of Fd-FNR to the grana pool of cytb_6f that operates in LET, while the stromal lamellae pool of cytb_6f, which is free to bind Fd-FNR, takes part in CET.
